# Targeting hyperactive TGFBR2 for treating MYOCD deficient lung cancer

**DOI:** 10.7150/thno.59816

**Published:** 2021-05-03

**Authors:** Qian Zhou, Wensheng Chen, Zhenzhen Fan, Zhipeng Chen, Jinxia Liang, Guandi Zeng, Lu Liu, Wanting Liu, Tong Yang, Xin Cao, Biao Yu, Meng Xu, Ye-Guang Chen, Liang Chen

**Affiliations:** 1 MOE Key Laboratory of Tumor Molecular Biology and Key Laboratory of Functional Protein Research of Guangdong Higher Education Institutes, Institute of Life and Health Engineering, College of Life Science and Technology, Jinan University, Guangzhou 510632, China.; 2 Zhongshan Hospital, Institute of Clinical Science, Fudan University Shanghai Medical College, 180 Fengling Road, Shanghai, 200032, China.; 3 State Key Laboratory of Bioorganic and Natural Products Chemistry, Center for Excellence in Molecular Synthesis, Shanghai, Institute of Organic Chemistry, University of Chinese Academy of Sciences, Chinese Academy of Sciences, 345 Lingling Road, Shanghai, 200032, China.; 4 Department of Oncology, The First Affiliated Hospital, Jinan University, Guangzhou 510632, China.; 5 The State Key Laboratory of Membrane Biology, Tsinghua-Peking Center for Life Sciences, School of Life Sciences, Tsinghua University, Beijing, China.; 6 Max-Planck Center for Tissue Stem Cell Research and Regenerative Medicine, Guangzhou Regenerative Medicine and Health Guangdong Laboratory, Guangzhou, China.

**Keywords:** tumor suppressor gene, targeted therapy, drug resistance

## Abstract

**Purpose:** Clinical success of cancer therapy is severely limited by drug resistance, attributed in large part to the loss of function of tumor suppressor genes (TSGs). Developing effective strategies to treat those tumors is challenging, but urgently needed in clinic.

**Experimental Design:** MYOCD is a clinically relevant TSG in lung cancer patients. Our *in vitro* and *in vivo* data confirm its tumor suppressive function. Further analysis reveals that MYOCD potently inhibits stemness of lung cancer stem cells. Mechanistically, MYOCD localizes to TGFBR2 promoter region and thereby recruits PRMT5/MEP50 complex to epigenetically silence its transcription.

**Conclusions:** NSCLC cells deficient of MYOCD are particularly sensitive to TGFBR kinase inhibitor (TGFBRi). TGFBRi and stemness inhibitor synergize with existing drugs to treat MYOCD deficient lung cancers. Our current work shows that loss of function of MYOCD creates Achilles' heels in lung cancer cells, which might be exploited in clinic.

## Introduction

De novo or acquired resistance significantly limits the clinical outcome of cancer treatments. There is increasing evidence that dysfunction of TSGs severely impacts response of cancer cells to chemotherapy 1, targeted therapy 2 and immunotherapy 3. There remains an urgent need to develop effective therapies for these resistant cancer patients.

Lung cancer is the leading cause of cancer-related deaths among all cancer types. There were an estimated 2.1 million new cases and 1.7 million deaths as of year 2018 globally 4. Gain-of-function mutations of driver genes are the main causes of tumorigenesis of lung cancer and have been relatively better characterized, with inhibitors against these oncoproteins approved for treating lung cancer patients in clinic. Impressive clinical success has been seen with targeted therapies against oncogenic mutant EGFR, ROS1, C-MET and EML4-ALK 5. Unfortunately, patients who initially responded to these therapies inevitably relapse. Moreover, a portion of patients show de novo resistance to these therapies. Partly due to these difficulties, the overall 5-year survival rate for lung cancer patient is still below 20% 6, 7.

Reports showed that inactivation of TSGs was responsible for a significant portion of drug resistance in current lung cancer therapy 2, 3, 8. The loss-of-function mutations of specific TSGs profoundly changes the signal network, which in turn changes the physiological behavior of cancer cells. For the purpose of successful therapy, TSGs remain to be systemically identified and characterized for lung cancers.

Myocardin (MYOCD) was cloned and verified to be both necessary and sufficient for the development and differentiation of most smooth muscle cell (SMC) in 2001 9. It is expressed specifically in contractile vascular, gastrointestinal and genitourinary smooth muscle and cardiac muscle tissues in adulthood as well as respective progenitor cells during embryonic development. Structure of MYOCD features multiple functionally distinct domains, including amino (N)-terminus, basic domain, extended amphipathic α-helix resembling a leucine zipper, stretch of glutamine (Q)-rich region, and SAP domain 10. MYOCD is a potent co-transcriptional activator, regulating the development and differentiation of cardiomyocyte and SMC lineages 9. Recent work revealed several functions unrelated to muscle development however, including facilitating embryonic vascular and postnatal development 11, repressing vascular inflammation, inhibiting VSMC dedifferentiation and proliferation 12-14, and regulating lipid metabolism 15.

The TGF-β signaling regulates various biological process, including cell apoptosis, differentiation 16, EMT 17, and proliferation^16^ ^18^. Canonical TGF-β signaling occurs when TGF-β ligand binds to TGFBR2, which then recruits and phosphorylates TGFBR1 to activate downstream SMAD2 and SMAD3. The phosphorylated SMAD2/3 then forms a complex with SMAD4 and translocated to the nucleus to regulate the transcription of TGF-β target genes 19.

Histone methylation is an epigenetic marker and plays a vital role in cell function. PRMT5 monomethylates and symmetric dimethylates arginine 20, importance of which is highlighted in a variety of cellular processes, including transcriptional regulation and germ cell development and several diseases. Studies have revealed that Grg4 complex is composed of PRMT5 and MEP50 and is essential for transcriptional repression mediated by PRMT5 and MEP50 21. Recently, crystal structure of the PRMT5/MEP50 complex has been resolved 22.

Here, we report that expression of MYOCD is downregulated NSCLC cancer samples in comparison to para-tumoral tissues and that its expression is positively correlated with overall survival rate. *In vitro* studies reveal potent suppressive role of MYOCD in colony formation of lung cancer cell lines in 2-D and soft-agar culture conditions. CRISPR/CAS9 mediated deletion of MYOCD significantly promoted the growth and development of lung cancer in mouse model of autochthonous NSCLC while lentivirus mediated overexpression significantly inhibited lung cancer development. We find that MYOCD negatively regulates stemness of lung cancer cells. Mechanistically, MYOCD localizes to TGFBR2 promoter region and thereby recruits PRMT5/MEP50 complex to epigenetically silence its gene transcription. Importantly, TGFBR kinase inhibitor and cancer stemness inhibitor synergize with existing drugs to treat lung cancer deficient of MYOCD.

## Results

### MYOCD is an essential TSG in lung cancer

**Figure 1:**
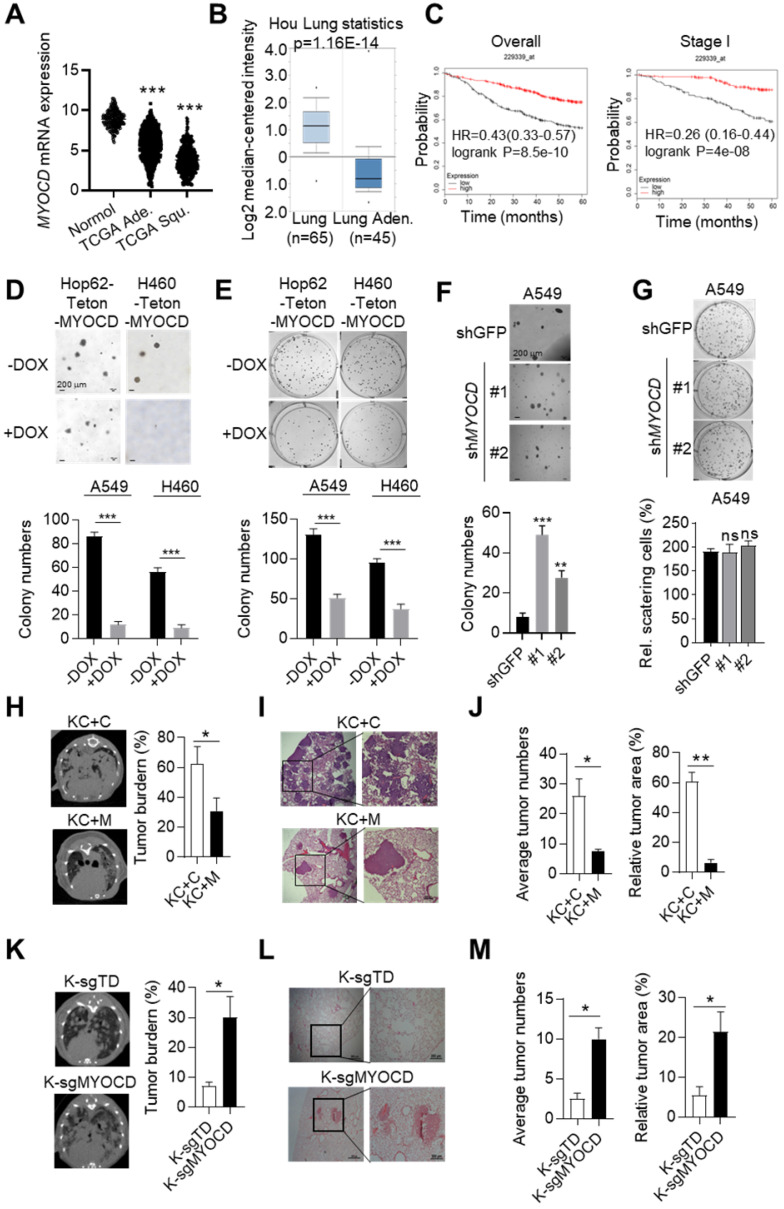
** MYOCD is an essential tumor suppressor in lung cancer.** (**A**) MYOCD mRNA expression in TCGA lung cancer tissue and GTEx lung tissue. The clinical database from UCSC Xena (http://xena.ucsc.edu/compare-tissue/) was used for analysis. (**B**) MYOCD mRNA expression in lung tissues and lung cancers from Oncomine database. Lung cancer expression data of Hou study was downloaded from Oncomine and used for analysis. (**C**) K-M survival analysis in NSCLC patients (http://kmplot.com/analysis/) (left: overall survival, right: stage I patients' survival). (**D-E**) MYOCD suppressed colony formation of Hop62 and H460 cell. Hop62-Teton-MYOCD (1000 cells) and H460-Teton-MYOCD cells (1000 cells) were respectively inoculated in soft agar in 6 well-plates (D) or directly in 6 well-plates **(E)** and treated with DOX or not treated for around 2 weeks before quantification for colonies. Statistics of colonies numbers (lower panel) and representative images (upper panel). (**F**) MYOCD knockdown promoted colony formation ability of A549 cells. A549 (1000 cells) were transfected with indicated shRNA plasmids for about 14 days before quantification or soft-agar colonies. Representative images of sphere assay (upper panel) and statistics of sphere formation (lower panel). (**G**) Effect of MYOCD knockdown on 2-D colony formation ability of A549 cells. A549 (1000 cells) were transfected with indicated shRNA plasmids for about 14 days before quantification colonies for 2-D plate. Representative images (upper panel) and statistics of colony formation (lower panel). (**H**) Overexpression of MYOCD slowed down the lung tumor initiation in KC mouse model. KC+C and KC+M mice were fed with DOX food for around12 weeks before CT scan. MRI images KC+C mice (n=3) and KC+M mice (n=3) of lung cancer bearing mice (left); Quantification of tumor burden (right). (**I**) Representative images of H&E staining of the lung tissues obtained from KC+C and KC+M mice. (**J**) Statistics of the average number of tumors (left) and relative tumor area (right). (**K**) MYOCD knockout promoted lung tumor initiation of lsl-Kras^G12D^ mice. MRI images of lung of K-sgTD mice (n=10) and K-sgMYOCD mice (n=10) of lung cancer bearing mice (left); Quantification of tumor burden (right). (**L**) Representative images of H&E staining of K-sgTD and K-sgMYOCD lung tissues. (**M**) Quantification of average tumor numbers (left panel) and relative tumor area (right panel).

Our previous *in-vivo* screening suggested that somatic knockout (KO) of MYOCD tended to promote tumor progression of Kras^G12D^ driven lung cancer 23. To find out whether MYOCD is a clinically relevant TSG, we analyzed its mRNA level in lung tumor tissues and para-tumoral tissues in TCGA database. Interestingly, our data suggested that the mRNA expression of MYOCD was dramatically lower in lung adenocarcinoma and squamous carcinoma samples than in paired normal tissues (Figure 1A). We also downloaded gene expression data from oncomine website (https://www.oncomine.org) and compared MYOCD expression level in lung cancers against para-tumoral tissues. Consistently, we found significantly lower expression of MYOCD mRNA levels in lung cancer samples (Figure 1B & S1A). We further analyzed TCGA data and found that the expression of MYOCD was positively associated with overall survival of lung cancer patients (Figure 1C, left). Of note, we also saw the same trend in stage I lung patients (Figure 1C, right), strongly suggesting that MYOCD was a clinically relevant TSG in lung cancer.

To further investigate the function of MYOCD in lung cancer, we set out to generate lung cancer cell lines for inducible overexpression or knockdown of MYOCD. We first checked MYOCD expression in various lung cancer cell lines through an online database (http://xenobase.crownbio.com). We found that A549 harbored relatively higher level of MYOCD mRNA level while H460 and HOP62 expressed lower level (Table S1), which is confirmed by qRT-PCR analysis (Figure S1B). We then generated Hop62 and H460 cell lines for DOXycycline (DOX) inducible expression of MYOCD (designated Hop62-Teton-MYOCD, H460-Teton-MYOCD respectively) (Figure S1C). Interestingly, we found that ectopic expression of MYOCD dramatically suppressed Hop62 and H460 cells to form colonies in soft-agar and 2D-plate cultures (Figure 1D-E). Conversely, knockdown of MYOCD with shRNA significantly enhanced the A549 cell to form colonies in soft-agar (Figure 1F & S1D). Of note, knockdown of MYOCD didn't significantly promote the A549 cell to grow colonies in 2-D plate (Figure 1G), suggesting that 2-D colony formation ability may not be a consistent phenotype on lung cancer cells elicited by MYOCD knockdown.

To further investigate its TSG function *in vivo*, we overexpressed MYOCD in pulmonary epithelia of lung cancer transgenic mice. For this purpose, we generated a cohort of Kras^G12D^/CC10rtTA transgenic mice 24 (designated KC, Figure S1E). KC mice develop lung cancers recapitulating human lung adenocarcinoma after feeding with DOX-containing diet (Dox-diet) for about 3 months, but remain tumor-free on normal diet 24. These KC mice were then infected with lentivirus containing Teton-MYOCD elements through intranasal instillation (designated KC+M) following protocol that we reported earlier 25.The KC+M mice started expressing MYOCD in lung after feeding DOX-diet for 48 hours (Figure S1F). In parallel, we infected KC mice with lentivirus packaged with vacant Teton-Puro vector as control cohort (Control group, KC+C) (Figure S1E-S1F). After 3 months of DOX-diet treatment, KC+C mice began to pant and showed a hunched posture, indicative of severe lung disease. Computed tomography imaging (CT) showed that KC+C mice had heavy tumor burdens in both lungs (Figure 1H). Pathological analysis revealed poorly differentiated lung adenocarcinoma with characteristics of diffuse bronchial adenocarcinoma. In sharp contrast, KC+M mice looked normal at this stage, and CT imaging showed a significantly lower lung cancer burden (Figure 1H). Consistently, pathological examination revealed significantly lighter tumor burdens in the lungs of these mice (Figure 1I-1J). These data strongly argued that overexpression of MYOCD inhibited the development of autochthonous lung cancers *in vivo*.

To model clinical settings of MYOCD deletion in lung cancer, we tested the impact of MYOCD knockout on lung cancer development using lsl-Kras^G12D^ transgenic mice, a model widely accepted to faithfully recapitulate the clinical course of lung cancer patients. Jacks and colleagues reported a protocol to simultaneously inactivate a target gene and activate mutant Kras in the lung compartment of these mice using recombinant lentivirus co-expressing Cre and CRISPR/CAS9 26. Using this protocol, we generated a cohort of lsl-Kras^G12D^ mice with lung epithelial knockout of MYOCD (K-sgMYOCD) and a control cohort targeting TdTomato (control group, K-sgTD). Western analysis revealed efficient elimination of MYOCD protein level in lungs of K-sgMYOCD mice (Figure S1G). CT imaging revealed that deletion of MYOCD significantly promoted Kras^G12D^-driven lung cancer progression (Figure 1K). We detected lung tumor nodules in 60% sgMYOCD mice in contrast to only around 30% K-sgTD mice post 16 weeks nasal inhalation of recombinant lentivirus. Pathological examination showed that deletion of MYOCD significantly increased the Kras^G12D^-driven lung tumor numbers and area (Figure 1L-1M).

Collectively, our data suggested that MYOCD was a potent and clinically relevant TSG in lung cancer.

### MYOCD inhibits stemness of lung cancer cell

TSGs exert their tumor suppressive functions frequently by inducing cell cycle arrest, senescence or apoptosis. FACS analysis showed no significant changes of apoptotic populations between DOX-treated Hop62-Teton-MYOCD cells and vehicle-treated control (Figure S2A), indicating that overexpression of MYOCD did not affect apoptosis in Hop62 cell. We also detected no significant difference of cell cycle distribution and senescence between DOX-treated and vehicle-treated Hop62-Teton-MYOCD cells (Figure S2B-S2D).

**Figure 2:**
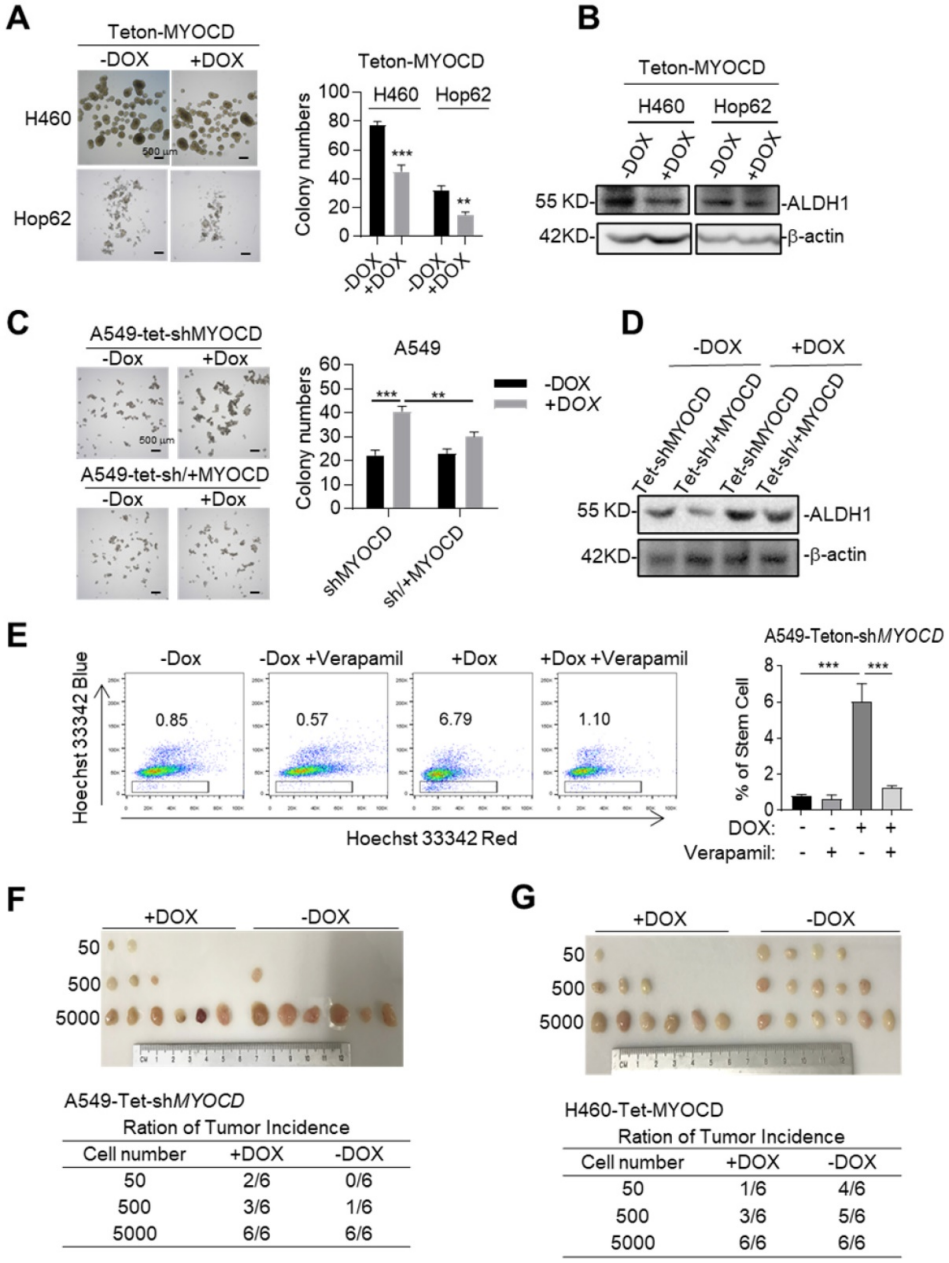
** MYOCD inhibits stemness of lung cancer cells.** (**A**) MYOCD inhibited sphere formation ability of H460 and Hop62 cells. H460-Teton-MYOCD and Hop62-Teton-MYOCD cells were treated with DOX or not treated for around 2 weeks before quantification. Representative images of sphere assay (left panel), statistics of sphere formation of colony numbers (right panel). (**B**) MYOCD reduced ALDH1 expression in H460 and Hop62 cells. H460-Teton-MYOCD and Hop62-Teton-MYOCD cells were not treated or treated with DOX for 48 hours. The whole lysates were analyzed by IB with the indicated antibodies. (**C**) MYOCD knockdown promoted sphere formation ability of A549 cells. Representative images of sphere assay (left), statistics of sphere formation of colony numbers (right). (**D**) MYOCD inactivation increased ALDH1 expression. (**E**) MYOCD inactivation increased frequency of side population cells in A549 cells. Side Population was analyzed through uptake of Hoechst33342 red with or without the presence of verapamil. Representative images of FACS analysis (left) and statistics of stem cells (right). (**F-G**) Tumor incidence was measured by Limiting Dilution Assays (LDA). A549-Teton-sh*MYOCD* cells (F) or H460-Teton-MYOCD (G) were subcutaneously injected 6-week-old female BALB/c nude mice, followed by treatment with DOX or control diet for 21 days before sacrificed mice. Images of tumor (upper panel), number of mice with a positive response (response = tumor >100 mm^3^) at 21 days post-injection (lower panel).

Cancer stem cells (CSCs) play a critical role in tumor development, relapse, and metastasis. Reports have shown that stemness of CSCs is negatively regulated by some TSGs 27, 28. We then wonder whether MYOCD functions as an inhibitor of stemness of lung cancer cells. To this end, we conducted sphere formation assay (SFA) to assess self-renewal potential of CSCs *in vitro* and found that overexpression of MYOCD reduced sphere-forming ability of H460 and Hop62 (Figure 2A). Consistently, we found ectopic expression of MYOCD inhibited expression of ALDH1, a CSC marker and functional player in solid tumors 29, in H460 and HOP62 cells (Figure 2B & S2D). Conversely, DOX induced MYOCD knockdown significantly promoted and re-expression of MYOCD suppressed A549 cells to form spheres (Figure 2C & S2E). In line with this, knockdown of MYOCD promoted the expression of ALDH1 and replenishing MYOCD compromised this effect (Figure 2D). Hoechst 33342, a DNA binding dye, can be pumped out by ABCG2, serving as the basis of side-population (SP) assay to identify CSCs in certain types of cancers 30. FACS analysis showed that MYOCD knockdown significantly increased percentage of SP in A549 cells (Figure 2E). Collectively, our results indicated that MYOCD inhibited stemness of lung CSCs.

We went on to test the ability of MYOCD to inhibit stemness of lung cancer on SCID mice through *in vivo* limiting dilution assay, a widely accepted method to determine the frequency of tumor initiating cell of an established cell line. 3 dilutions of A549-Teton-sh*MYOCD* cells (50 to 5,000) were subcutaneously inoculated in a replica set of BALB/c Nude Mice, followed by feeding control or DOX-diet. After 21 days of treatment, we saw no significant difference in tumor incidence at high concentrations (5,000 cells). In contrast, while 3 of 6 sites (50%) of 500 inoculum and 2 of 6 sites (33%) of 50 inoculum in cohort of DOX treatment (knockdown of MYOCD expression) developed tumor, control-diet fed cohort developed at a significantly lower incidence (1 of 6 sites (17%) and 0 of 6 sites respectively) (Figure 2F). Conversely, MYOCD overexpression significantly inhibited tumor incidence of H460-Teton-MYOCD cell in DOX-treated nude mice (Figure 2G).

Studies have revealed correlation between Epithelial-Mesenchymal Transition (EMT) and stemness of cancer cells 31, 32. We noticed that MYOCD knockdown shifted A549 cells from cobble-stone like towards a more elongated spindle-like shape, a morphology characteristic of EMT (Figure S2F). In line with this, we found that knockdown of MYOCD reduced expression of CDH1 (E-cadherin), an epithelial marker, and increased expression of mesenchymal markers like Vimentin and SNAIL and SLUG (Figure S2G). Moreover, MYOCD knockdown significantly enhanced the migrating ability in wound-healing assay (Figure S2H) and transwell assay (Figure S2I).

Taken together, these results consistently showed that MYOCD is an inhibitor of stemness of lung cancer cells.

### MYOCD inhibits lung cancer stemness through suppressing TGFBR signaling

**Figure 3:**
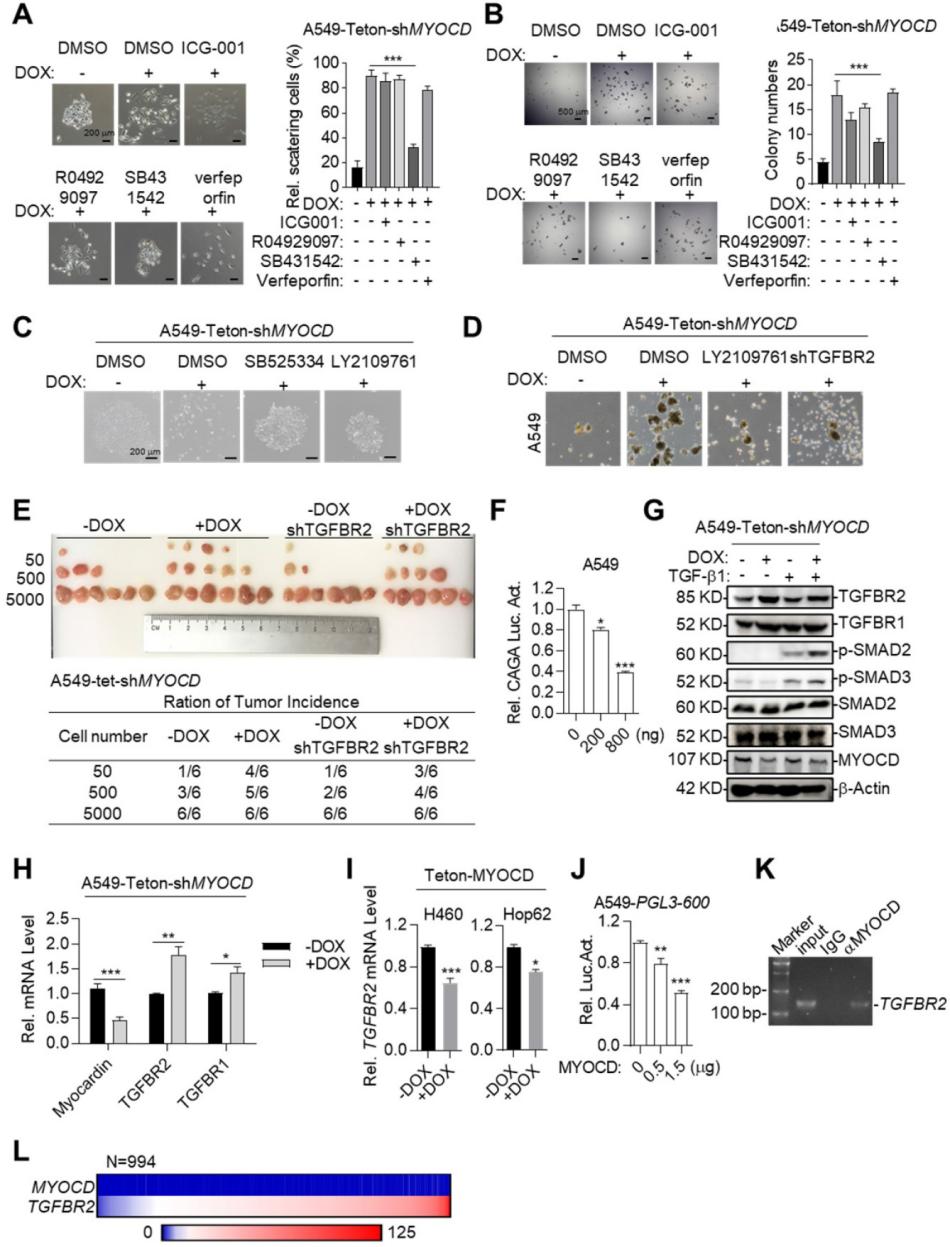
** MYOCD inhibits lung cancer stemness through suppressing TGFBR signaling.** (**A-B**) SB431542 significantly inhibited EMT activity and sphere forming ability induced by MYOCD knockdown. A549-Teton-sh*MYOCD* cells were treated or untreated with DOX for 2 days, and subsequently incubated with indicated drugs (ICG001: 1 μM, R04929097: 1 μM, SB431542: 1 μM, verfeporfin: 0.5 μM) for around 10 days before quantification scattering of cell clones **(A)** and sphere assay **(B)**. (**C**) MYOCD knockdown leading to lung cancer cells scattering was inhibited by TGF-β signaling inhibitors. Representative images of colonies of A549-Teton-sh*MYOCD*. A549-Teton-sh*MYOCD* (200 cells) were left untreated or treated with DOX for 48 hours, and subsequently with DMSO and TGF-β signaling pathway inhibitors including SB525334 (1 µM) and LY2109761 (300 nM) for 10 days before photographing. (**D**) MYOCD inactivation mediated sphere formation was suppressed by LY2109761 and TGFBR2 shRNA. Representative images of spheres of A549-Teton-sh*MYOCD*. A549-Teton-sh*MYOCD* were infected or not infected with DOX-inducible TGFBR2 shRNA and subsequently treated with/without DOX for 2 days later, then the cells were treated with DMSO and LY2109761 (300 nM) for 10 days before quantification. (**E**) Tumor incidence was measured by LDA. A549-Teton-sh*MYOCD* were infected or not infected with DOX-inducible TGFBR2 shRNA, then indicated cells were inoculated subcutaneously in the flank of 6-week-old female BALB/c nude mice fed with DOX diet. Images of tumor (upper panel), number of mice with a positive response (response = tumor >100 mm^3^) at 30 days post-injection (lower panel). (**F**) MYOCD inhibited SMAD2/3luciferase activity in A549 cell in does dependent manner. A549 cell was transfected with pGL3-(CAGA)_12_ luciferase reporter plasmid (0.8 μg), increased amounts of MYOCD expression plasmid (0, 0.4, 0.8 μg) and 100 ng of renilla luciferase plasmid, followed by monitoring luciferase 48 hours later. (**G**) Knockdown of MYOCD elevated phospho-SMAD2, phospho-SMAD3 expression and increased TGFBR2 expression. A549-Teton-sh*MYOCD* cells were not treated or treated with DOX for 3 days, followed by treatment with TGF-β for 3 hours. Whole cell lysates (WCL) were analyzed by immunoblots with the indicated antibodies. (**H**) MYOCD inactivation promoted *TGFBR2* transcription in lung cancer cells. Total RNA was extracted from A549-Teton-sh*MYOCD* was treated with DOX for 2 days or not treated w. The expression of the indicated genes was quantified through qPCR. (**I**) MYOCD suppressed TGFBR2 transcription in lung cancer cells. RNA was extracted from H460/Hop62-Teton-MYOCD treated without or with DOX for 2 days. TGFBR2 expression was checked with qPCR. (**J**) MYOCD reduced TGFBR2 luciferase activity in A549 cells. A549 cells were transfected with pGL3-600 reporter plasmid (0.8 μg), increased amounts of MYOCD expression plasmid (0, 0.5, 1.5 μg) and 100 ng Renial-luc plasmid, followed by monitoring luciferase 48 hours later. (**K**) MYOCD localized in TGFBR2 promoter region. ChIP-PCR analysis of the *TGFBR2* promoters was performed using antibodies against MYOCD in A549 cells. (**L**) Reverse correlation between expression level of MYOCD and TGFβR2 in clinical lung cancer samples downloaded from Proteinatlas (https://www.proteinatlas.org/).

We next asked the molecular mechanisms underlying cancer-stemness suppressive role of MYOCD. We first systemically assayed the impact of inhibition of pathways commonly involved in stemness on A549-Teton-sh*MYOCD* cell. To this end, we administered inhibitors against WNT, TGF-β, Notch and YAP pathway and carefully assayed EMT features of cell clones in 2-D plate and sphere forming ability in A549-Teton-sh*MYOCD* cells. Strikingly, we found that SB431542 (TGF-β signaling pathway inhibitor) significantly inhibited EMT activity induced by MYOCD knockdown (Figure 3A). Consistently, SB431542 significantly inhibited sphere forming ability enhanced by MYOCD knockdown (Figure 3B). We found that another two TGFBR inhibitors (SB525334 and LY2109761) inhibited EMT at a similar degree on A549-Teton-sh*MYOCD* (Figure 3C & S3A). More importantly, TGFBR2 knockdown (Figure S3C) inhibited the stemness of both A549-Teton-sh*MYOCD* to a similar degree as LY2109761 did in SFA *in vitro* (Figure 3D & S3B) and tumor formation assays *in vivo* (Figure 3E). We also noticed that MYOCD dose-dependently inhibited TGF-β pathway luciferase reporter in A549 cells (Figure 3F), indicating inhibition of TGF-β pathway activity. Collectively, our results indicated that MYOCD expression downregulated stemness of lung CSCs through inhibiting TGFBR signaling pathway.

To further study how MYOCD negatively regulates TGF-β signaling pathway, we checked impact of MYOCD knockdown on phosphorylation status of signaling elements in TGF-β pathway. Western analysis revealed that knockdown of MYOCD elevated phospho-SMAD2 and phospho-SMAD3 expression (Figure 3G). Interestingly, qRT-PCR and western blot analysis showed that MYOCD knockdown upregulates TGFBR2 expression through mRNA transcription (Figure 3G-3H). Conversely, ectopic expression of MYOCD suppressed mRNA level of TGFBR2 in Hop62 and H460 cells (Figure 3I). These results suggested that MYOCD downregulated TGFBR2 at transcription level. Of note, we also observed that MYOCD knockdown slightly increased TGFBR1. However, it was not as drastic as TGFBR2. Therefore, we focused our further experimental efforts on TGFBR2 regulation.

Supporting our conclusion, we found that MYOCD dose-dependently inhibited TGFBR2 promoter activity revealed by luciferase reporter assay (Figure 3J). Furthermore, Chromatin immunoprecipitation followed by PCR (ChIP-PCR) analysis revealed that MYOCD localized in TGFBR2 promoter region (Figure 3K).

Of note, we found that MYOCD mRNA level in clinical samples was almost universally low as reflected by Fragments Per Kilobase of transcript per Million (FPKM) in RNA-sequencing data of 994 lung cancer samples analyzed (FPKM<1 in 98% patients). In contrast, 83% patients expressed high level of TGFBR2 (FPKM>10) in this cohort. Due to uneven distribution of expression value in this cohort, we were not able to reach statistical significance for correlation between expression levels of TGFBR2 and MYOCD. Nevertheless, our data suggested the clinical relevance of our observation (Figure 3L).

Taken together, our data showed that MYOCD inhibited lung cancer cell stemness through inhibiting TGFBR2 transcription.

### MYOCD recruits PRMT5/MEP50 methyltransferase complex to TGFBR2 promoter region

**Figure 4:**
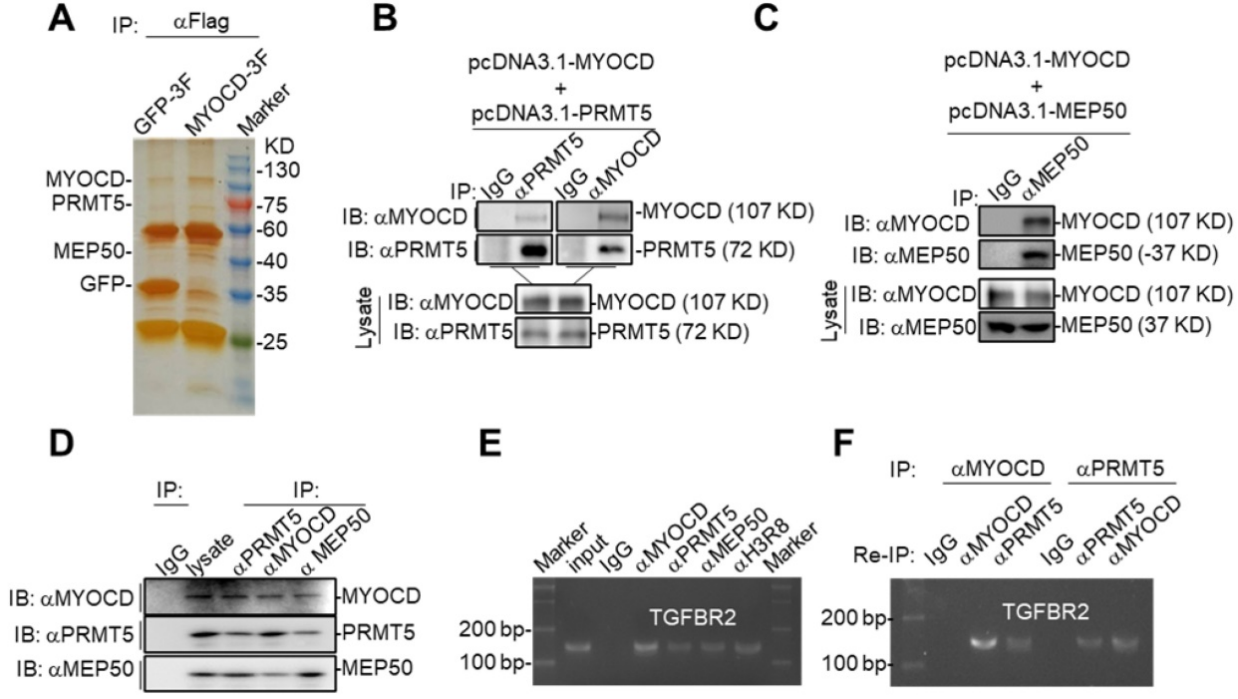
** MYOCD recruits PRMT5/MEP50 methyltransferase complex to TGFBR2 promoter region.** (**A**) Silver-staining of SDS-PAGE separated protein samples immunoprecipitated with antibody against Flag from 293T-GFP 3×FLAG and 293T-MYOCD 3×FLAG cells. (**B-C**) MYOCD is associated with PRMT5 and WDR77. The 293T cells were transfected with the indicated plasmids (2.5 μg each). Coimmunoprecipitation and immunoblot were performed with the indicated antibodies. (**D**) Endogenous MYOCD is associated with PRMT5 and WDR77 in A549 cells. Coimmunoprecipitation experiments were performed with indicated antibodies, and the immunoprecipitates and WCL were analyzed with the indicated antibodies. (**E-F**) MYOCD, PRMT5, WDR77 and H3R8mes localized in TGFBR2 promoter region. **(E)** ChIP-PCR analysis of the *TGFBR2* promoters was performed using indicated antibodies in A549 cells; **(F)** Re-ChIP analysis of the *TGFBR2* promoters was performed using antibodies against indicated antibodies in A549 cells.

In our study, we found that MYOCD inhibited transcription of TGFBR2 in lung cancer cells, which is contrast to popular findings that MYOCD is a transcription activator 33. In order to elucidate the molecular mechanism underlying its transcriptional inhibitory activity, we checked the binding partners of MYOCD immunoprecipitated from 293T cells ectopically overexpressing FLAG-tagged MYOCD. Silver staining of the SDS-PAGE gel clearly revealed distinct protein species in MYOCD pull-down samples compared to IgG-enriched control (Figure 4A). Liquid chromatography-mass spectrometry (LC-MS) analysis of MYOCD enriched protein samples identified 71 specific protein species (Table S2). Among the binding partners, PRMT5 and MEP50 caught our attention, as PRMT5/MEP50 complex is involved in symmetrically methylating arginine residues of histones to regulate gene expression 34.

We hypothesized that MYOCD recruited PRMT5/MEP50 complex to TGFBR2 promoter to modify histones in this region such that TGFBR2 transcription was silenced in A549 cells. When PRMT5 or MEP50 was respectively co-overexpressed with MYOCD in HEK293 cells, MYOCD efficiently pulldown PRMT5 or MEP50 (Figure 4B-4C). In order to find out whether these 3 proteins form a complex in NSCLC cells, we also managed to conduct co-IP experiments on lysate derived from 5 × 10^7^ A549 cells. In this experiment, we found that antibody against any one protein immunoprecipitated the other two (Figure 4D). Taken together, these data strongly argued that MYOCD, PRMT5 and MEP50 function as a protein complex in NSCLC cells.

PRMT5 and MEP50 form a protein methyltransferase complex that symmetrically dimethylates H2AR3, H3R2, H4R3, and H3R8 34, all of which activate or suppress gene transcription in a context-dependent manner. We then checked whether PRMT5/ MEP50 bound the same region of TGFBR2 promoter as MYOCD did. Our ChIP-PCR assay revealed that MYOCD, PRMT5 and MEP50 co-occupied the TGFBR2 promoter region (Figure 4E). More importantly, H3R8mes was detectable in the same region (Figure 4E). To solidify the notion that MYOCD recruited the PRMT5/MEP50 complex to regulate TGFBR2 transcription, we conducted ChIP-Re-ChIP experiments. In these experiments, fragmented chromatins were first immunoprecipitated with antibodies against MYOCD or PRMT5. The immunoprecipitates were then reciprocally re-immunoprecipitated with anti-PRMT5 or anti-MYOCD and DNA were extracted from the secondary immunoprecipitates for quantifying the amount of TGFBR2 promoter region. Our ChIP-Re-ChIP results solidly showed that MYOCD, PRMT5 and MEP50 co-occupied the same TGFBR2 promoter region (Figure 4F).

### MYOCD recruits PRMT5/MEP50 methyltransferase complex to epigenetically silence TGFBR2 transcription

**Figure 5:**
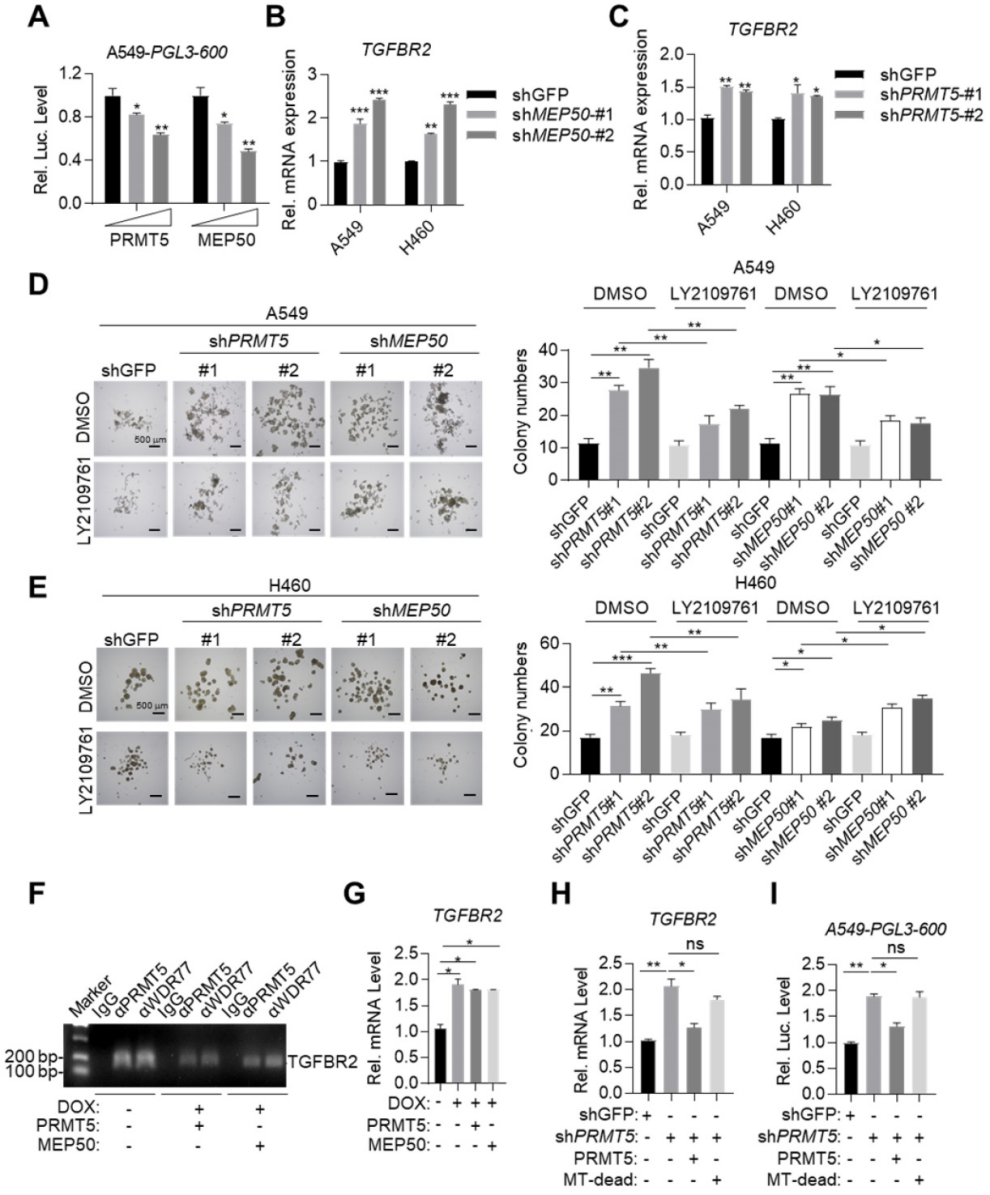
** MYOCD recruits PRMT5/MEP50 methyltransferase complex to epigenetically silence TGFBR2 transcription.** (**A**) PRMT5 and WDR77 reduced TGFBR2 luciferase activity in A549 cells in does dependent manner. A549 cells were transfected with pGL3-600 reporter plasmid (0.8 μg), increased amounts of PRMT5 or WDR77 expression plasmid (0, 0.5, 1.5 μg) and 100 ng renilla luciferase plasmid, followed by monitoring luciferase 48 hours later. (**B-C**) PRMT5 and WDR77 inactivation promoted TGFBR2 transcription in A549 and H460 cells. RNA was extracted from indicated cell lines; qPCR was performed to check the expression of TGFBR2. (**D-E**) PRMT5 and WDR77 knockdown promoted sphere forming ability and this ability was suppressed by LY2109761 in A549 (D) and H460 (E) cells. Cells were treated with DMSO or LY2109761 (300 nM) for 10 days before quantification. Representative images of sphere assay (left) and statistics of sphere formation (right). (**F**) PRMT5/WDR77 associates with TGFBR2 is dependent on MYOCD. ChIP-PCR analysis of TGFBR2 promoters using indicated antibodies in A549 cells. (**G**) PRMT5/WDR77 failed to suppress *TGFBR2* transcription in A549 MYOCD inactivation cells. A549-Teton-sh*MYOCD* cells were transfected with indicated plasmids, then the cells were left treated or untreated with DOX for 2 days, RNA was extracted and qPCR was performed to check the expression of *TGFBR2*. (**H**) PRMT5 methyltransferase activity was important for MYOCD mediated suppression of TGFBR2 transcription. The A549 cells were firstly transfected with a control or sh*PRMT5* plasmid (2 μg). cells were selected with puromycin (1 μg/ml) for 24 h, then cells were re-transfected with PRMT5 or PRMT5 MT-dead expression plasmid (0.3 μg each). RNA was extracted to analysis *TGFBR2* mRNA level 48 hours after re-transfection. (**I**) PRMT5 methyltransferase mutation failed to activate TGFBR2 reporter activity. The A549 cells were firstly transfected with a control or sh*PRMT5* plasmid (2 μg). Cells were selected with puromycin (1 μg /ml) for 24 h and then re-transfected with the *PGL-600* promoter reporter and PRMT5 or PRMT5 MT-dead expression plasmid (0.3 μg each). Luciferase assays were performed 48 h after re-transfection.

We asked whether PRMT5/MEP50 complex was recruited by MYOCD to epigenetically silence TGFBR2 transcription. Ectopic expression of PRMT5 or MEP50 in A549 lung cancer cells dose-dependently inhibited *TGFBR2* promoter reporter activity (Figure 5A). Conversely, knockdown of *PRMT5* or *WDR77* (encoding MEP50) increased *TGFBR2* mRNA expression (Figure 5B-5C & S4). We also found that knockdown of *PRMT5* or *WDR77* enhanced sphere forming ability of A549 and H460 cells, which was inhibited by TGFBR inhibitor LY2109761 (Figure 5D-5E). Furthermore, ChIP experiment revealed that PRMT5 and MEP50 no longer occupied the *TGFBR2* promoter region in A549 cells with MYOCD knockdown (Figure 5F). These results suggested PRMT5/MEP50 complex and MYOCD function as a functional entity to inhibit TGFBR2 transcription.

We also found that overexpressed PRMT5 and MEP50 failed to further reduce *TGFBR2* mRNA expression level in DOX treated A549-Teton-sh*MYOCD* cells (Figure 5G), consistent with our conclusion that MYOCD recruited PRMT5/MEP50 complex to the TGFBR2 promoter region to regulate its transcription. PRMT5/MEP50 complex symmetrically dimethylates H3R8, which activates or suppresses gene transcription. Deletion (360 aa- 372 aa) mutant has been reported to eliminate the methyltransferase activity of PRMT5 (designated MT-dead). We then re-expressed MT-dead or wild-type PRMT5 respectively in A549-sh*PRMT5* cells and found that wild-type PRMT5, but not MT-dead mutant, inhibited the *TGFBR2* transcription (Figure 5H-5I). Collectively, our data solidly showed that MYOCD recruited PRMT5/MEP50 methyltransferase complex and epigenetically modified TGFBR2 promoter region to silence its transcription in lung cancer cells.

### Targeting TGFBR and stemness synergizes with existing drug to treat MYOCD-deficient lung cancers

Our above data suggested that MYOCD inactivation led to TGFBR2 hyperactivation and enhanced stemness of lung cancer cell. We then checked whether inhibitors against stemness or TGFBR could synergize with current therapeutics for treating MYOCD-deficient lung cancers. Potent inhibitors targeting TGF-β signaling are now under clinical trial (NCT03143985 & NCT04037514 @clinicaltrials.gov).

Mutant Kras positive lung cancers lack effective treatment options in clinic. We wonder whether our work could be translated into therapy against this subtype of lung cancer. Of note, we revealed that around 32% of Kras mutation positive lung cancer patients concurrently had low expression of MYOCD (designated K^+^/M^-^ patients, Table S3). As A549 harbored a mutant Kras, we then chose DOX treated A549-Teton-sh*MYOCD* cells to model this portion of patients and tested treatment on this cells line.

**Figure 6:**
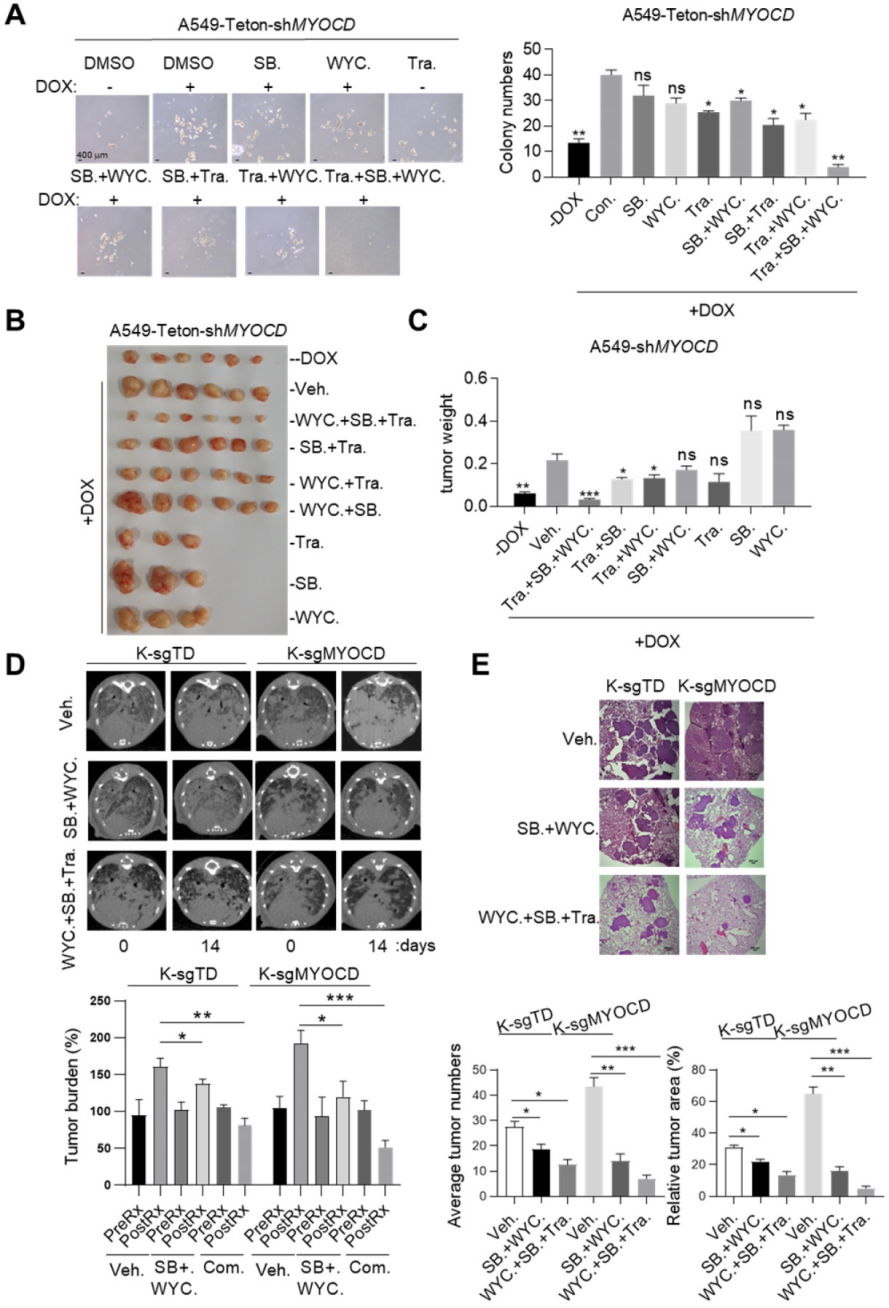
** Targeting TGFBR and stemness synergizes with existing drug to treat MYOCD-deficient lung cancers.** (**A**) Effects of SB525334, WYC209 or Trametinib treatments alone or combination on MYOCD inactivated A549 cells. A549-Teton-sh*MYOCD* cells were treated with DOX to knockdown MYOCD expression. Cells were simultaneously treated with DMSO, SB525334, WYC209, Trametinib alone or combination treatments for 10 days before quantification. Representative images of sphere assay (left panel) and statistics of sphere formation (right panel). (**B-C**) Weights of tumors in xenograft mouse models treated with indicated drugs. A549-Teton-sh*MYOCD* cells (2× 10^6^) were implanted subcutaneously into the flank of 6-week-old female BALB/c nude mice. When the tumors reached a volume of around 100 mm^3^, animals were randomized into 9 groups (n = 6 per group or n=3 per group) for treatments with indicated drugs for around 14 days before sacrificed. (**D-E**) SB525334 (1 mg/kg/day), WYC209 (1 mg/kg/day) synergizes with trametinib (1 mg/kg/day) to shrink lung tumor in KRAS^G12D^/MYOCD^-/-^ mice. **(D)** MRI image of lung of K-sgTD and K-sgMYOCD mice treated with vehicle (Veh.) or combination-treatment (SB. + WYC.; WYC. + SB. + Tra.) (upper panel) and quantification of tumor burden of MRI image (lower panel). **(E)** Representative images of H&E staining of the lung tissues obtained from Veh. and Com. group from KRAS^G12D^/MYOCD^-/-^ mice (upper panel), statistics of tumor numbers and tumor area (lower panel).

Earlier, we reported that MEK1/2 inhibitor treatment led to partial regression of mutant Kras driven lung cancer 35. We have also identified a CSC targeting reagents, WYC209 36. We then set out to treat K^+^/M^-^ lung cancer with combination of inhibitors for MEK1/2, TGFBR2, and CSC stemness. However, MEK inhibitors and TGFBR inhibitors are all known to be toxic, which limited their application at high concentration in clinic 37, 38. We first treated A549-Teton-sh*MYOCD* cells with SB525334, WYC209 and Trametinib, an FDA approved MEK1/2 inhibitor, at relatively low concentrations. Interestingly, we found that while singlet treatment minimally reduced sphere forming ability, combination of either two partially inhibited sphere forming ability. Strikingly, combination of all 3 inhibitors most potently inhibited sphere forming ability (Figure 6A). Importantly, we observed similar trend of treatment effect in mice harboring A549-Teton-sh*MYOCD* derived xenograft tumors (Figure 6B-6C). Of note, combination of these 3 drugs is well tolerated by mice as indicated by the constant weight of mice during treatment (Figure S5). Collectively, these results indicated that combination of SB525334 and WYC209 sensitized K^+^/M^-^ lung cancer to trametinib inhibition.

Having confirmed the efficacy and safety of combination of SB525334, WYC209 and Trametinib in treating K^+^/M^-^ lung cancers in xenograft mouse model, we then went on to test this treatment scheme on transgenic mouse model of autochthonous lung cancer. For this purpose, we generated a cohort of Kras^G12D^/MYOCD^-/-^ (K-sgMYOCD, see Figure 1K-M for details) transgenic mice and randomized them for treatments with above combinational scheme after lung cancers were documented by CT scanning. Strikingly, CT scanning indicated significant tumor shrinkage in mice treated with this combination for 14 days (Figure 6D). Pathological examination revealed that in the lung of combination treated mouse, residual tumor nodules harbored obvious thickening of alveolar wall and fibrosis, indicating the healing process (Figure 6E). In line with our hypothesis, we found that K-sgTD mice (lsl-Kras G12D mice knockout for TdTomato, serving as control see Figure 1K-M) were relatively less sensitive to this treatment (Figure 6D-E). Taken together, our results suggested that TGFBR inhibitors and CSC stemness inhibitors may synergize with current drug for treating MYOCD-deficient lung cancers.

## Discussion

Currently, targeted therapy is the first line therapy for NSCLC patients. However, de novo drug resistance and acquired drug resistance severely limit the success of these otherwise efficacious therapies. Our current work showed that loss of function of TSG rewired signaling of a tumor cell, and thereby created an Achiel's heal. In the case of MYOCD, loss of function of this important TSG leads to hyperactivation of TGFBR. We have shown that TGFBR inhibitors exhibited exquisite cytotoxicity to NSCLC cells with loss of function of MYOCD, which could be exploited in lung cancer therapy.

MYOCD primarily functions as a transcriptional coactivator through interacting with SMAD3 directly, which promotes TGFB signaling pathway in the smooth muscle 39 and enhances TGFB-induced EMT and metastasis of NSCLC 40. However, transcription of MYOCD has been reported to be frequently down regulated in mesenchymal cells, resulting in disruption of their differentiation and at last their malignant transformation 41. The ability of MYOCD to inhibit tumor growth were reported in various cancer types 42, 43.

Inactivation of MYOCD could be adaptive response of the NSCLC cancer cells to selection of targeting reagents. However, our analysis of TCGA data has also shown that inactivation of MYOCD is an early event in development of lung cancer. Given its ability to negatively regulate TGF-β signaling, MYOCD inactivation may be a spontaneous event during tumor development, but not a drug selected event. Indeed, TGF-β signaling exerts tumor suppressive role in early phase of tumorigenesis, but exerts tumor promoting role in late phase. Cancer cells evolved an intricate system to turn TGFBR-SMAD signaling from tumor suppressive in early phase to oncogenic in late phase of tumor development 44.

When considering tumor microenvironment, TGF-β could be provided by many tumorigenic cell components, including M2 type macrophages 45, Tregs 46, and myeloid-derived suppressor cell (MDSC) 47. Earlier, we have shown that inaction of other tumor suppressor genes in NSCLC can also lead to hyperactivation of TGFBR-SMAD signaling axis 25. In the light of tumor immunotherapy, TGFBR inhibition not only sensitizes NSCLC cells to targeting therapies, but normalizes immunity against tumors.

Cancer stem cells (CSC) are critical for tumor development, relapse and drug resistance 48. CSC is the minority of cell population within tumor that are hardest to target. Fortunately, recent intensive research have revealed unique features of biology of CSCs, which could provide targeting opportunities: GPX4 is critical for maintaining CSC state 49; HIF transcription factor plays an important role for survival of CSCs 50; potassium channel protein involves in maintaining CSCs 51. Modifying the function of these proteins is expected to create opportunities for treating NSCLCs. Earlier, we have reported a CSC targeting small molecule, WCY209 36. In our current work, we validated the notion that targeting this population could synergize with existing drug for treating NSCLC tumors.

In our study, we have solidly shown that MYOCD recruits PRMT5/MEP50 histone methyltransferase complex to TGFBR2 gene promoter region, and thereby epigenetically silences transcription of TGFBR2. Currently, small molecular inhibitors have been developed targeting epigenetic reads and writers. It will be interesting to test whether these reagents could synergistically treat NSCLC patients with MYOCD deficiency with existing drugs in clinic.

In summary, we put forward an idea that loss-of-function mutation of TSGs creates Achilles' heel, which could be exploited for cancer targeted therapy.

## Materials and Methods

### Constructs, reagents and antibodies

The pLVX-Tetone-Puro plasmid (Clontech), pLVX-Tetone-Puro-MYOCD (Clontech), psPAX2 (Addgene), pMD2.G (Addgene) and pLKO.1-puro (Addgene); DOX (Sigma); FBS (Gibco), DMEM/F12 medium (Gibco), RPMI medium 1640 (Gibco) and DMEM (Gibco); Western blotting substrate (Millipore); B27 (Invitrogen); dual-specific luciferase assay kit was from Promega; FGF and EGF were purchased from PeproTech; RIPA lysis buffer was from Santa Cruz; protease and phosphatase inhibitor cocktail (Roche); Super Nova CT (SNC-100, PINSENG HEALTHCARE); Lipofectamine 3000 was from Invitrogen; Antibodies against ZEB1, ZEB2, TWIST, SNAIL, SLUG, E-Cadherin, Vimentin, TGFBR1, TGFBR2, SMAD2, SMAD3, phospho-SMAD2, phospho-SMAD3 (Cell Signaling Technology); HA and β-actin (Sigma); ALDH1, PRMT5, WDR77, MEP50 (Abcam); A549 (ATCC), NCI-H460 (ATCC), Hop62 (ATCC), HEK293 (ATCC); BALB/c nude mice were purchased from Beijing Vital River Laboratory Animal Technology Co., Ltd.; Protein A/G Agarose beads was obtained from Thermo Scientific; silver staining Rapid silver staining kit's was purchased from Beyotime; SB525334 (Selleck chemicals), Trametinib (Selleck chemicals); WYC209 was provided by Biao Yu. shMYOCD#1: TRCN0000151657; shMYOCD#2: TRCN0000150565; shPRMT5#1: TRCN0000107086; shPRMT5#2: TRCN0000107088; shWDR77#1: TRCN0000072781; shWDR77#2: TRCN0000072782 (sigma shRNA library); pcDNA3.1-MYOCD, pcDNA3.1-PRMT5, pcDNA3.1-WDR77, all truncations and point mutations were constructed by standard molecular biology techniques.

### Colony formation assay

1×10^3^ cells were seeded in 6 well plate with 10% FBS and 1% P/S. After around 2 weeks culture without or with DOX incubation, the cells were fixed with 4% formaldehyde, then stained with crystal violet.

### Sphere formation assay

1×10^3^ cells were seeded in Ultra-Low attachment 6 well plate in serum-free conditioned medium containing DMEM/F12 medium, 20 µL/mL B27 supplement, 20 ng/mL basic FGF and 20 ng/mL EGF. After around 2 weeks culture, the spheres with the diameter over 100 µm were counted.

### Soft agar assay

The bottom agar (1.2%) with 10% FBS and 1% P/S was plated in 6 well plate for 1 hour in cell culture hood. Then the upper layer of agar (0.6%) containing the cell suspensions (1×10^3^) in RPMI-1640 medium with 10% FBS and 1% P/S was plated. The cells were cultured around 14 days with or without DOX treatments before imaging and colonies counting.

### Protein extraction and immunoblotting

The cells were lysed with RIPA lysis buffer, then the concentration was measured with Bradford assay. Around 30 μg protein were loaded for SDS-PAGE. Immunoblot analysis were performed as previously described 52.

### Transgenic model mouse care and use

Animal experiments were approved by the Institutional Committee for Animal Care and Use at Jinan University (20200902-04). All animal work was performed in strict accordance with the approved protocol.

All mice were housed in a pathogen-free environment in Jinan University. For transgenic **K**ras^G12D^/**C**C10rtTA mice, Lentivirus with MYOCD or puro were delivered to mouse lung intranasally. After 1 week, the mice were fed with DOX food until the day of CT scan and sacrifice.

For transgenic lsl-Kras^G12D^ mice, which were infected with recombinant lentivirus co-expressed Cre and CAS9 to infect lsl-Kras^G12D^ mice by intravenous injection. According to manufactures' protocol, mouse was scanned by Super Nova CT. Collected mouse lungs for H&E staining. In order to quantify the tumor burden in transgenic mice, we calculated the relative tumor area and tumor number of all tumor regions in the H&E section under the microscope.

### Tumorigenicity *in vivo* xenograft and limiting dilution assay

Cells were counted and resuspended at 5 × 10^3^, 500 and 50 cells in mixture of equal volumes of PBS and Matrigel were implanted subcutaneously into the flank of 6-week-old female BALB/c nude mice. Tumor diameters were observed weekly until the mice were sacrificed after 21 days of observation. Tumor volume (cm^3^) = D×d^2^/2, (D is the longest, d is the shortest diameter). The number of mice with a positive response were counted (response = tumor > 0.1 cm^3^) at around 3 weeks post-injection.

### RNA extraction and real-time RT-PCR (qRT-PCR)

Total RNA was extracted using Trizol reagent, and real-time PCR analysis was performed to measure the mRNA level of the indicated genes. The data shown is the relative abundance of the indicated mRNA normalized to GAPDH. The gene-specific primer sequence is as follows:

- TGFBR1-F: ACAAAGCAGAGCCCATCTGT;
- TGFBR1-R: GCTGCTCCTCCTCGTGCT;
- TGFBR2-F: GTCTGTGGATGACCTGGCTAAC;
- TGFBR2-R: GACATCGGTCTGCTTGAAGGAC;
- PRMT5-F: CTAGACCGAGTACCAGAAGAGG;
- PRMT5-R: CAGCATACAGCTTTATCCGCCG;
- WDR77-F: CTCAGGTCACTTGTGTTGCTGC;
- WDR77-R: ATCTGTGATGCTGGCTTGGGAC;
- MYOCD-F: GCAACACCGATTCAGCTACCTAG;
- MYOCD-R: GGTATTGCTCAGTGGCGTTGAAG.

### Chromatin immunoprecipitation (ChIP)-PCR

A549 cells were seeded in eight 15 cm dishes, the cells were cross-linked with 1% formaldehyde and sonicated using the following parameters: 5 seconds on, 10 seconds off, 15 cycles at 25% set power. Divided the total cell lysate into several aliquots and mix with the indicated antibodies, then 50 μL Protein A/G Sepharose beads, and the other was mixed with normal IgG and 50 μL Sepharose beads. After overnight incubation, the beads were washed with ChIP wash buffer. The chromatin was eluted, the crosslink was reversed, and the DNA was extracted with phenol/chloroform. Finally, the eluted DNA was dissolved in ddH2O before further experiments.

The following sequences were used for TGFBR2 ChIP-PCR:

- Forward-CTCGCTCAATTTCTTGTGATGTCCAGTG;
- Reverse-TGGGTTAAGCGCATTCTAGTTGGAGG.

### Silver staining and Mass spectrometry assays

Silver staining and Mass spectrometry assays were performed as described before 52. In brief, perform silver staining according to the instructions of the Silver staining Rapid silver staining kit's description. Cutting the target strip for further analysis. Then of the In-gel digestion was performed and the products of In-gel digestion were used for Mass spectrometry analysis. Mass spectrometry assays were performed on ABI 5600 Triple TOF.

### Transfection and luciferase assay

A549 cells were transfected by lipofectamine 3000. Add control plasmids to ensure that each transfection received the same amount of DNA. To normalize for transfection efficiency, pRL-TK reporter plasmid was added to each transfection. 24-48 hours after transfection, use a dual-specific luciferase assay kit for luciferase assay.

### Histopathological analysis

Lung tissue samples were fixed in 10% neutral buffered formalin, dehydrated, and embedded in paraffin. Samples were subsequently sectioned at 5 µm thickness and stained with hematoxylin and eosin (H&E) for histopathology.

### Statistical analysis

Statistics were performed using GraphPad Prism 7.04, Student's t-test was used to compare differences between two experimental groups. Data are presented as means ± SD and error bars denote SD; n=3; *P < 0.05; **P < 0.01; ***P < 0.001.

## Supplementary Material

Supplementary figures and methods.

Table S1.

Table S2.

Table S3.
