# Supplementary material for: Targeting hyperactive TGFBR2 for treating MYOCD deficient lung cancer
Source: Theranostics. 2021 May 3;11(13):6592–606. doi: 10.7150/thno.59816 (PMC8120205; doi:10.7150/thno.59816)
Supplement: Supplementary file 1 — Supplementary figures and methods. [file thnov11p6592s1.pdf]

Title: Targeting hyperactive TGFBR2 for treating MYOCD deficient lung cancer

Running Title: TGFBR2 inhibition for MYOCD low NSCLC

Qian Zhou[1*#], Wensheng Chen[1*], Zhenzhen Fan[1*], Zhipeng Chen[1*], Jinxia Liang[1*], Guandi Zeng[1], Lu Liu[1], Wanting Liu[1], Tong Yang[1], Xin Cao[2], Biao Yu[3], Meng Xu[4], Ye-Guang Chen[5,6#], Liang Chen[1#]

[1] MOE Key Laboratory of Tumor Molecular Biology and Key Laboratory of Functional Protein Research of Guangdong Higher Education Institutes, Institute of Life and Health Engineering, College of Life Science and Technology, Jinan University, Guangzhou 510632, China;

[2] Zhongshan Hospital, Institute of Clinical Science, Fudan University Shanghai Medical College, 180 Fengling Road, Shanghai, 200032, China;

[3] State Key Laboratory of Bioorganic and Natural Products Chemistry, Center for Excellence in Molecular Synthesis, Shanghai, Institute of Organic Chemistry, University of Chinese Academy of Sciences, Chinese Academy of Sciences, 345 Lingling Road, Shanghai, 200032, China;

[4] Department of Oncology, The First Affiliated Hospital, Jinan University, Guangzhou 510632, China;

[5] The State Key Laboratory of Membrane Biology, Tsinghua-Peking Center for Life Sciences, School of Life Sciences, Tsinghua University, Beijing, China;

[6] Max-Planck Center for Tissue Stem Cell Research and Regenerative Medicine, Guangzhou Regenerative Medicine and Health Guangdong Laboratory, Guangzhou, China

[*] These authors contributed equally to this work

[#] Corresponding Author:

Qian Zhou, Telephone: (086)-020-8522-6223, Email: zhouqian_whu@163.com

Ye-Guang Chen, Telephone: (086)-010-6278-4794, Email: ygchen@tsinghua.edu.cn

Liang Chen, Telephone: (086)-020-8522-2875, Email: chenliang@jnu.edu.cn

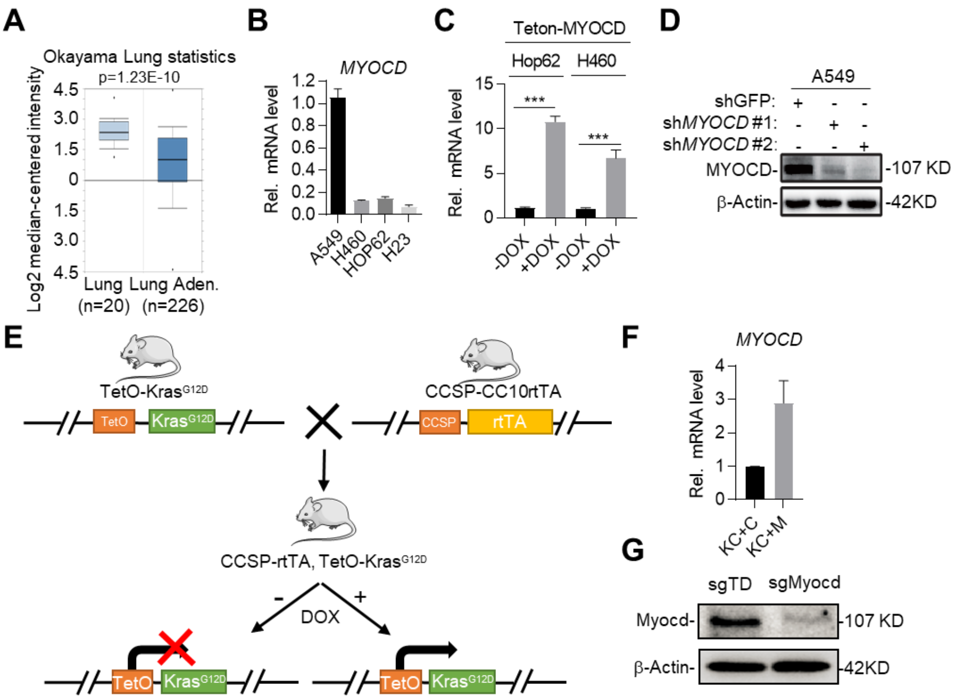

Figure S1. MYOCD is an essential tumor suppressor in lung cancer

(**A**) MYOCD mRNA expression in lung tissues and lung cancers from Oncomine database. Lung cancer expression data of Okayama study was downloaded from Oncomine and used for analysis. (**B**) MYOCD mRNA expression in the indicated lung cancer cell lines. (**C**) Construct Hop62-Teton-MYOCD and H460-Teton-MYOCD stable cell line. Hop62-Teton-MYOCD and H460-Teton-MYOCD cells were not treated or treated with DOX for 2 days, total RNA was extracted to analysis the *MYOCD* mRNA Level. (**D**) Knockdown of MYOCD in A549 cells. A549 cells were transfected with shGFP or two independent MYOCD-shRNA plasmids (2 μg). After 24 hours, cells were selected with puromycin (1 μg/ml) for 72 hours, WCL were analyzed by indicated antibodies. (**E**) Schematic representations of **Kras**^{G12D}/**CC10rtTA** (KC) mice. (**F**) MYOCD expression level in lung of KC mice. Lung from KC+C mice or KC+M mice was extracted RNA to analysis MYOCD mRNA expression after feeding DOX-diet for 48 hours. (**G**) MYOCD expression level in lung of lsl-KrasG12D transgenic mice.

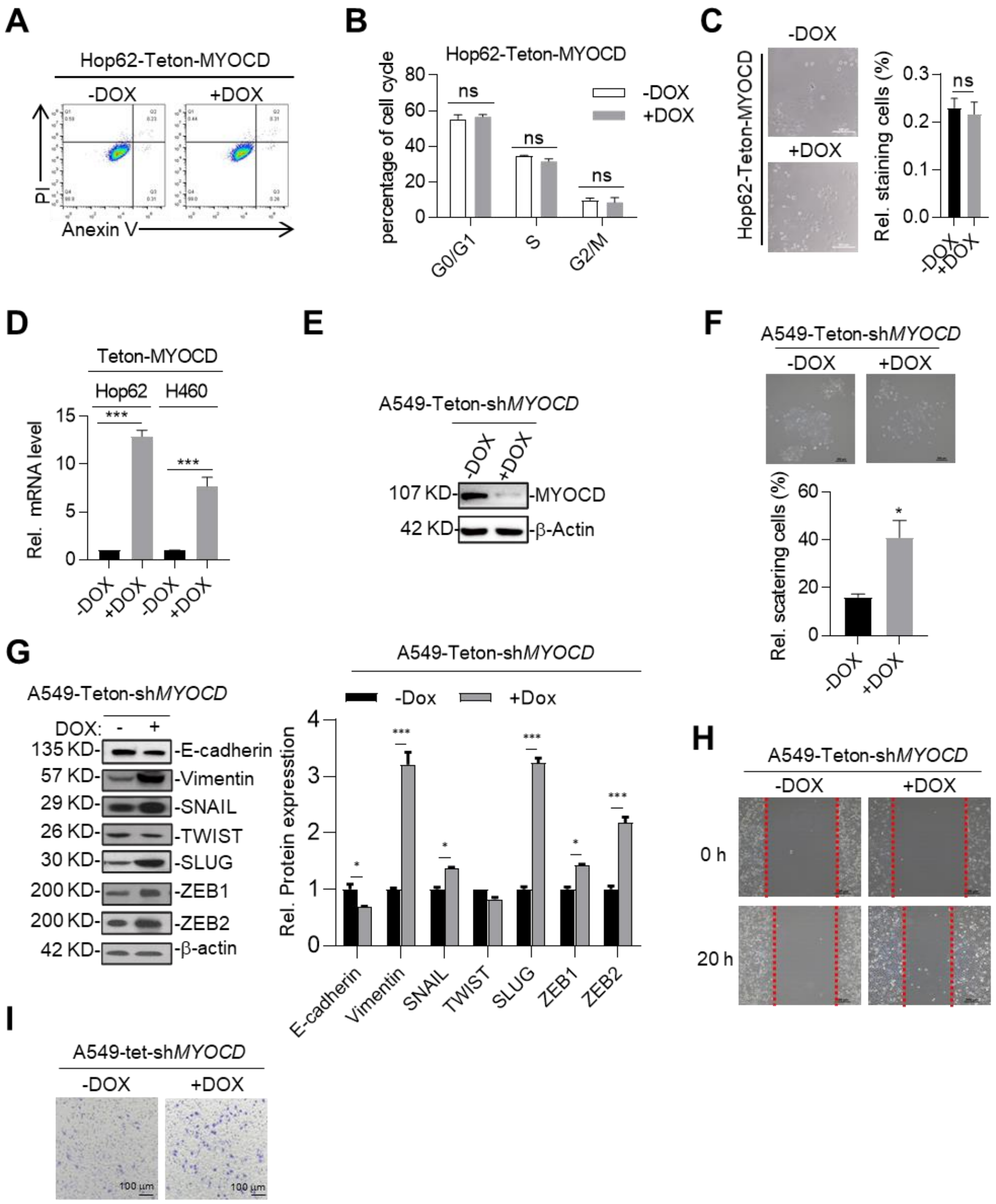

Figure S2. MYOCD inhibits stemness of lung cancer cells

(**A**) MYOCD did not affect the apoptosis of Hop62 cells. Hop62-Teton-MYOCD were not treated or treated with DOX for 2 days before FACS assay. **(B)** MYOCD does not affect cell cycle distribution in Hop62 cells. Hop62-Teton-MYOCD were not treated or treated with DOX for 2 days before cell cycle analysis. **(C)** Representative image of senescence-associated β-galactosidase staining. Hop62-Teton-MYOCD cells were treated with or without DOX (1 μg/mL) for 48 hours. (**D**) Generation of H460/Hop62-Teton-MYOCD cell line. H460/Hop62-Teton-MYOCD cells were not treated or treated with DOX for 2 days, total RNA was extracted to analysis the *MYOCD* mRNA Level.

(**E**) Effects of DOX induced expression of MYOCD in A549-Teton-sh*MYOCD* cell. A549-Teton-sh*MYOCD* cells were not treated or treated with DOX for 2 days, then WCL were analyzed by immunoblots with the indicated antibodies. (**F**) MYOCD knockdown leading to lung cancer cells scattering. Representative images of colonies of A549-Teton-sh*MYOCD* cells (upper panel) and statistic of scattering cells (lower panel). (**G**) Analysis of epithelial, mesenchymal markers and EMT drivers in A549 cells. The indicated cells were were not treated or treated with DOX for 2 days, WCL were analyzed by immunoblots with the indicated antibodies (left panel), right panel showed statistics analysis of relative protein expression. (**H**) In vitro migration assay in A549-Teton-sh*MYOCD* cells. A549-Teton-sh*MYOCD* cells were not treated or treated with DOX for 2 days, then cells were scratched. Images show wound size at time 0 h and 20 h after scratch. (**I**) Transwell assays in A549-Teton-sh*MYOCD* cells. The migrated cells were stained and counted.

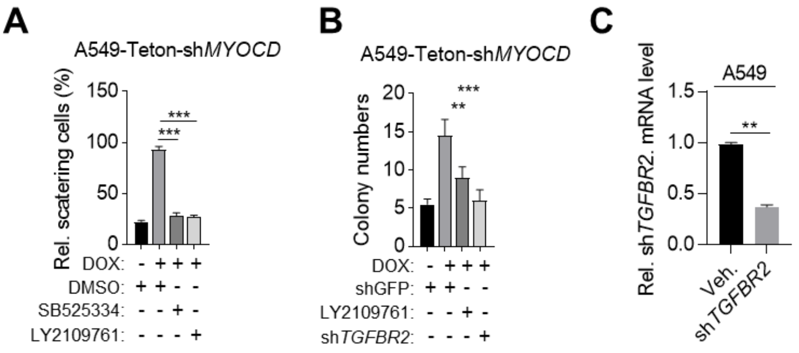

Figure S3. MYOCD inhibits lung cancer stemness through suppressing TGFBR signaling

(**A**) MYOCD knockdown leading to lung cancer cells scattering was inhibited by TGF-β signaling inhibitors. A549-Teton-sh*MYOCD* (200 cells) were not treated or treated with DOX for 2 days, DMSO and TGF-β signaling pathway inhibitors including SB525334 (1 μM) and LY2109761 (300 nM) were treated for the 10 days. Quantification of scattering cells. (**B**) MYOCD inactivation mediated lung cancer cells sphere formation was suppressed by LY2109761 and TGFBR2 shRNA. A549-Teton-sh*MYOCD* was infected or not infected with DOX-inducible TGFBR2 shRNA, then cells were left untreated or treated with DOX, 48 hours later, DMSO and LY2109761 (300 nM) were treated for 10 days. Quantification of sphere numbers. (**C**) Effects of DOX induced expression of TGFBR2 in A549-Teton-sh*TGFBR2* cell. RNA was extracted from A549-Teton-MYOCD cells, which were not treated or treated with DOX for 2 days. Expression of TGFBR2 was quantified through qPCR.

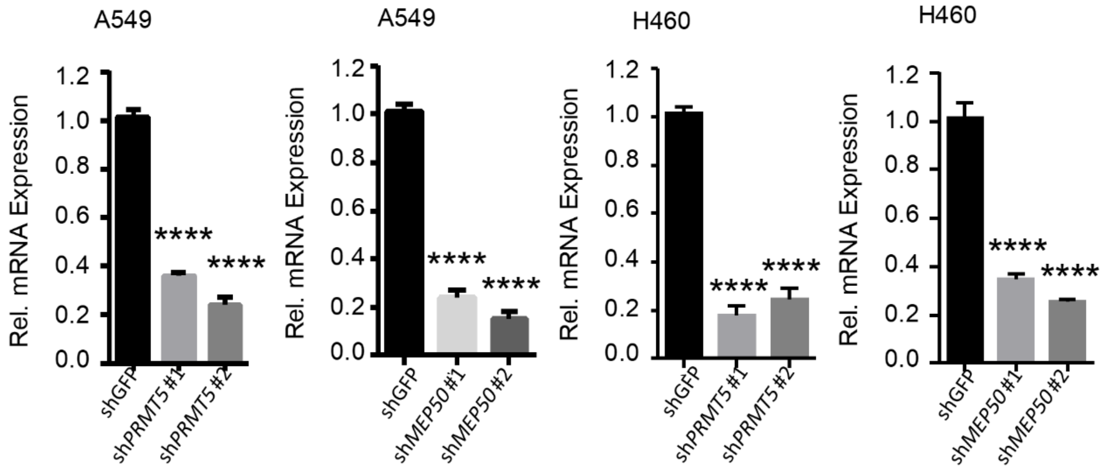

Figure S4. Knockdown of PRMT5 or WDR77 in A549 and H460 cells. A549 and H460 cells were transfected with shGFP or two independent PRMT5/WDR77-shRNA plasmids (2 μg). 24 hours after, cells were selected with puromycin (1 μg/ml) for 72 h, qPCR were performed after puro selection. Data are presented as means ± SD and error bars denote SD; n=3; *P < 0.05; **P < 0.01; ***P < 0.001; ***P < 0.0001.

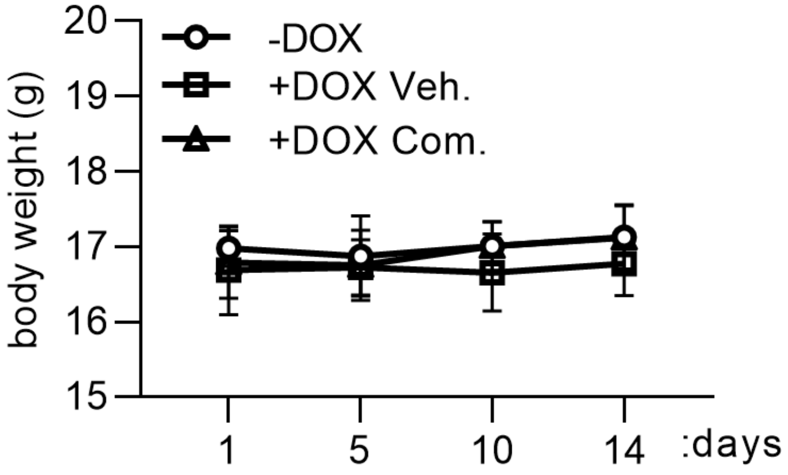

Figure S5. MYOCD expression and combination therapy do not affect the body weight of the mice. A549-Teton-sh*MYOCD* cells (2× 10^6) were implanted subcutaneously into the flank of BALB/c nude mice. When the tumors reached a volume of around 100 mm^3, animals were randomized into 9 groups (n = 6 per group or n=3 per group), then mice received different treatment as indicated in Figure 6B for around 14 days before sacrificed. Body weight of the mice (-DOX group, +DOX Veh. group & +DOX Com. group) was recorded as indicated time points.

Supplementary Materials and Methods

Generation of engineered cell lines

The HEK293 cells were transfected with psPAX2 and pMD2.G and lentiviral plasmids respectively. After 24 hours, the cells were incubated with the new medium for another 24 hours. Filter the medium containing the recombinant virus and add it to the designated cells in the presence of polybrene (8 μg/ml). Before conducting other experiments, select infected cells with puromycin (1μg/mL).

Cell cycle analysis

The cells were collected at 48 h after DOX treatment and fixed with 70% ethanol at -20°C overnight. According to the manufacturer's instructions, the cell cycle kit was used for cell cycle analysis. BD Accuri™ C6 flow cytometer was for FACS.

Senescence associated β-galactosidase staining assay

β-galactosidase staining was performed using the Cell Signaling Senescence β-Galactosidase Staining Kit according to manufacturer’s instruction. In brief, 0.2 M indicated cells were cultured 6 well plates with different treatments before staining. For statistics of β-galactosidase staining positive cells, the blue positive cells were counted with ImageJ.

Scratch wound healing assay

Indicated cells were cultured in 56 well plate, when cells were around 100% confluent, a scratch was made across the plates using a pipette’s tip. Cells were cultured and imaged as indicated time points to monitor the wound healing process.

Table 1:MYOCD expression in lung cancer cell Gene Expression-RNAseq

GENE	ID	CELL LINE	CANCER TYPE	LOG2(FPKM)	Source
MYOCD	ENSG00000141052	SQ1	Lung	-2	CCLE
MYOCD	ENSG00000141052	NCIH2085	Lung	-2	CCLE
MYOCD	ENSG00000141052	NCI-H1869	Lung	-2	CCLE
MYOCD	ENSG00000141052	DMS454	Lung	-2	CCLE
MYOCD	ENSG00000141052	NCIH647	Lung	-2	CCLE
MYOCD	ENSG00000141052	NCI-H1838	Lung	-2	CCLE
MYOCD	ENSG00000141052	NCIH2196	Lung	-2	CCLE
MYOCD	ENSG00000141052	NCI-H1703	Lung	-2	CCLE
MYOCD	ENSG00000141052	NCIH2347	Lung	-2	CCLE
MYOCD	ENSG00000141052	NCI-H2110	Lung	-2	CCLE
MYOCD	ENSG00000141052	NCIH1341	Lung	-2	CCLE
MYOCD	ENSG00000141052	HCC1195	Lung	-2	CCLE
MYOCD	ENSG00000141052	BEN	Lung	-2	CCLE
MYOCD	ENSG00000141052	NCI-H596	Lung	-2	CCLE
MYOCD	ENSG00000141052	NCIH2444	Lung	-2	CCLE
MYOCD	ENSG00000141052	NCI-H1299	Lung	-2	CCLE
MYOCD	ENSG00000141052	NCI-H69	Lung	-2	CCLE
MYOCD	ENSG00000141052	NCIH28	Lung/Pleura	-2	CCLE
MYOCD	ENSG00000141052	NCIH1755	Lung	-2	CCLE
MYOCD	ENSG00000141052	NCI-H1395	Lung	-2	CCLE
MYOCD	ENSG00000141052	EBC-1	Lung	-2	CCLE
MYOCD	ENSG00000141052	NCI-H211	Lung	-2	CCLE
MYOCD	ENSG00000141052	NCI-H1793	Lung	-2	CCLE
MYOCD	ENSG00000141052	NCIH2029	Lung	-2	CCLE
MYOCD	ENSG00000141052	PC14	Lung	-2	CCLE
MYOCD	ENSG00000141052	HCC1171	Lung	-2	CCLE
MYOCD	ENSG00000141052	NCI-H1648	Lung	-2	CCLE
MYOCD	ENSG00000141052	HCC1588	Lung	-2	CCLE
MYOCD	ENSG00000141052	HCC33	Lung	-2	CCLE
MYOCD	ENSG00000141052	NCI-H522	Lung	-2	CCLE
MYOCD	ENSG00000141052	NCI-H526	Lung	-2	CCLE
MYOCD	ENSG00000141052	LU99	Lung	-2	CCLE
MYOCD	ENSG00000141052	NCI-H2081	Lung	-2	CCLE
MYOCD	ENSG00000141052	NCIH854	Lung	-2	CCLE
MYOCD	ENSG00000141052	CORL279	Lung	-2	CCLE
MYOCD	ENSG00000141052	NCIH1184	Lung	-2	CCLE
MYOCD	ENSG00000141052	NCIH510	Lung	-2	CCLE
MYOCD	ENSG00000141052	NCI-H2452	Lung/Pleura	-2	CCLE
MYOCD	ENSG00000141052	NCIH1694	Lung	-2	CCLE
MYOCD	ENSG00000141052	COLO668	Lung	-2	CCLE
MYOCD	ENSG00000141052	CORL105	Lung	-2	CCLE
MYOCD	ENSG00000141052	NCI-H1105	Lung	-2	CCLE
MYOCD	ENSG00000141052	COR-L23	Lung	-2	CCLE
MYOCD	ENSG00000141052	DMS 53	Lung	-2	CCLE
MYOCD	ENSG00000141052	NCI-H1666	Lung	-2	CCLE
MYOCD	ENSG00000141052	A-427	Lung	-2	Crownbio
MYOCD	ENSG00000141052	NCI-H2009	Lung	-2	CCLE
MYOCD	ENSG00000141052	NCI-H2227	Lung	-2	CCLE

MYOCD	ENSG00000141052	NCI-H889	Lung	-2	CCLE
MYOCD	ENSG00000141052	DMS 79	Lung	-2	CCLE
MYOCD	ENSG00000141052	NCI-H1792	Lung	-2	CCLE
MYOCD	ENSG00000141052	DV-90	Lung	-2	CCLE
MYOCD	ENSG00000141052	NCI-H146	Lung	-2	CCLE
MYOCD	ENSG00000141052	NCI-H1688	Lung	-2	Crownbio
MYOCD	ENSG00000141052	NCI-H810	Lung	-2	CCLE
MYOCD	ENSG00000141052	NCI-H2122	Lung	-2	CCLE
MYOCD	ENSG00000141052	CAL12T	Lung	-2	CCLE
MYOCD	ENSG00000141052	ABC1	Lung	-2	CCLE
MYOCD	ENSG00000141052	NCIH2106	Lung	-2	CCLE
MYOCD	ENSG00000141052	NCI-H226	Lung/Pleura	-2	CCLE
MYOCD	ENSG00000141052	MsTo 211H	Lung/Pleura	-2	CCLE
MYOCD	ENSG00000141052	LK-2	Lung	-2	CCLE
MYOCD	ENSG00000141052	HCC1833	Lung	-2	CCLE
MYOCD	ENSG00000141052	LCLC-97TM1	Lung	-2	CCLE
MYOCD	ENSG00000141052	NCI-H1437	Lung	-2	CCLE
MYOCD	ENSG00000141052	LU65	Lung	-2	CCLE
MYOCD	ENSG00000141052	NCIH2171	Lung	-2	CCLE
MYOCD	ENSG00000141052	NCI-H1963	Lung	-2	CCLE
MYOCD	ENSG00000141052	SK-LU-1	Lung	-2	CCLE
MYOCD	ENSG00000141052	NCI-H1435	Lung	-2	Crownbio
MYOCD	ENSG00000141052	ACCMESO1	Lung/Pleura	-2	CCLE
MYOCD	ENSG00000141052	KNS62	Lung	-2	CCLE
MYOCD	ENSG00000141052	NCI-H1435	Lung	-2	CCLE
MYOCD	ENSG00000141052	T3M10	Lung	-2	CCLE
MYOCD	ENSG00000141052	NCI-H69	Lung	-2	Crownbio
MYOCD	ENSG00000141052	LUDLU1	Lung	-2	CCLE
MYOCD	ENSG00000141052	NCI-H2066	Lung	-2	CCLE
MYOCD	ENSG00000141052	PC-9	Lung	-2	Crownbio
MYOCD	ENSG00000141052	NCI-H1993	Lung	-2	Crownbio
MYOCD	ENSG00000141052	NCI-H226	Lung/Pleura	-2	Crownbio
MYOCD	ENSG00000141052	SW1573	Lung	-2	CCLE
MYOCD	ENSG00000141052	NCI-H1650	Lung	-2	Crownbio
MYOCD	ENSG00000141052	SHP-77	Lung	-2	Crownbio
MYOCD	ENSG00000141052	NCI-H522	Lung	-2	Crownbio
MYOCD	ENSG00000141052	NCI-H2228	Lung	-2	Crownbio
MYOCD	ENSG00000141052	NCI-H838	Lung	-2	CCLE
MYOCD	ENSG00000141052	NCI-H1666	Lung	-2	Crownbio
MYOCD	ENSG00000141052	NCI-H1975	Lung	-2	Crownbio
MYOCD	ENSG00000141052	NCI-H2087	Lung	-2	CCLE
MYOCD	ENSG00000141052	HCC827	Lung	-2	Crownbio
MYOCD	ENSG00000141052	HCC4006	Lung	-2	Crownbio
MYOCD	ENSG00000141052	NCI-H1395	Lung	-2	Crownbio
MYOCD	ENSG00000141052	HCC2935	Lung	-2	Crownbio
MYOCD	ENSG00000141052	NCI-H1417	Lung	-2	Crownbio
MYOCD	ENSG00000141052	SBC5	Lung	-2	CCLE
MYOCD	ENSG00000141052	HCC44	Lung	-2	CCLE
MYOCD	ENSG00000141052	IALM	Lung	-2	CCLE
MYOCD	ENSG00000141052	RERFLCAD2	Lung	-2	CCLE

MYOCD	ENSG00000141052	NCI-H1568	Lung	-2	CCLE
MYOCD	ENSG00000141052	EBC-1	Lung	-2	Crownbio
MYOCD	ENSG00000141052	Calu-1	Lung	-2	CCLE
MYOCD	ENSG00000141052	SK-MES-1	Lung	-2	CCLE
MYOCD	ENSG00000141052	JL1	Lung/Pleura	-2	CCLE
MYOCD	ENSG00000141052	NCI-H2073	Lung	-2	CCLE
MYOCD	ENSG00000141052	HCC2279	Lung	-2	CCLE
MYOCD	ENSG00000141052	NCI-H209	Lung	-2	CCLE
MYOCD	ENSG00000141052	NCI-H1650	Lung	-2	CCLE
MYOCD	ENSG00000141052	NCI-H446	Lung	-2	Crownbio
MYOCD	ENSG00000141052	DMS 53	Lung	-2	Crownbio
MYOCD	ENSG00000141052	NCI-H441	Lung	-2	CCLE
MYOCD	ENSG00000141052	NCI-H322	Lung	-2	CCLE
MYOCD	ENSG00000141052	NCI-H460	Lung	-2	CCLE
MYOCD	ENSG00000141052	NCIH1734	Lung	-2	CCLE
MYOCD	ENSG00000141052	NCI-H358	Lung	-2	CCLE
MYOCD	ENSG00000141052	SW1271	Lung	-2	CCLE
MYOCD	ENSG00000141052	NCI-H1155	Lung	-2	CCLE
MYOCD	ENSG00000141052	NCI-H1975	Lung	-2	CCLE
MYOCD	ENSG00000141052	NCI-H841	Lung	-2	CCLE
MYOCD	ENSG00000141052	NCI-H82	Lung	-2	CCLE
MYOCD	ENSG00000141052	NCIH2291	Lung	-2	CCLE
MYOCD	ENSG00000141052	SCLC21H	Lung	-2	CCLE
MYOCD	ENSG00000141052	NCIH196	Lung	-2	CCLE
MYOCD	ENSG00000141052	HCC827	Lung	-2	CCLE
MYOCD	ENSG00000141052	NCIH650	Lung	-2	CCLE
MYOCD	ENSG00000141052	NCIH524	Lung	-2	CCLE
MYOCD	ENSG00000141052	LC1F	Lung	-2	CCLE
MYOCD	ENSG00000141052	HCC2108	Lung	-2	CCLE
MYOCD	ENSG00000141052	HCC1359	Lung	-2	CCLE
MYOCD	ENSG00000141052	NCI-H460	Lung	-2	Crownbio
MYOCD	ENSG00000141052	HOP92	Lung	-2	CCLE
MYOCD	ENSG00000141052	HARA	Lung	-2	CCLE
MYOCD	ENSG00000141052	SW900	Lung	-2	CCLE
MYOCD	ENSG00000141052	NCI-H1573	Lung	-2	CCLE
MYOCD	ENSG00000141052	NCIH1092	Lung	-2	CCLE
MYOCD	ENSG00000141052	NCI-H2009	Lung	-2	Crownbio
MYOCD	ENSG00000141052	DMS 153	Lung	-2	CCLE
MYOCD	ENSG00000141052	NCI-H292	Lung	-2	CCLE
MYOCD	ENSG00000141052	LCLC-103H	Lung	-2	CCLE
MYOCD	ENSG00000141052	NCI-H2228	Lung	-2	CCLE
MYOCD	ENSG00000141052	CORL95	Lung	-2	CCLE
MYOCD	ENSG00000141052	NCIH1693	Lung	-2	CCLE
MYOCD	ENSG00000141052	CORL311	Lung	-2	CCLE
MYOCD	ENSG00000141052	HCC2935	Lung	-2	CCLE
MYOCD	ENSG00000141052	Calu-3	Lung	-2	CCLE
MYOCD	ENSG00000141052	NCI-H820	Lung	-2	Crownbio
MYOCD	ENSG00000141052	NCI-H1581	Lung	-2	CCLE
MYOCD	ENSG00000141052	NCIH1339	Lung	-2	CCLE
MYOCD	ENSG00000141052	RERFLCAD1	Lung	-2	CCLE

MYOCD	ENSG00000141052	CORL47	Lung	-2	CCLE
MYOCD	ENSG00000141052	NCI-H446	Lung	-2	CCLE
MYOCD	ENSG00000141052	SNU-2535	Lung	-2	Crownbio
MYOCD	ENSG00000141052	NCI-H1563	Lung	-2	CCLE
MYOCD	ENSG00000141052	NCI-H3255	Lung	-2	CCLE
MYOCD	ENSG00000141052	HCC-78	Lung	-2	CCLE
MYOCD	ENSG00000141052	NCIH1385	Lung	-2	CCLE
MYOCD	ENSG00000141052	NCI-H1876	Lung	-2	CCLE
MYOCD	ENSG00000141052	HCC1438	Lung	-2	CCLE
MYOCD	ENSG00000141052	HOP62	Lung	-2	CCLE
MYOCD	ENSG00000141052	NCI-H1573	Lung	-2	Crownbio
MYOCD	ENSG00000141052	NCI-H1781	Lung	-2	CCLE
MYOCD	ENSG00000141052	HCC95	Lung	-2	CCLE
MYOCD	ENSG00000141052	NCIH2342	Lung	-2	CCLE
MYOCD	ENSG00000141052	NCI-H82	Lung	-2	Crownbio
MYOCD	ENSG00000141052	ISTMES2	Lung/Pleura	-2	CCLE
MYOCD	ENSG00000141052	SHP-77	Lung	-2	CCLE
MYOCD	ENSG00000141052	NCI-H2052	Lung/Pleura	-2	CCLE
MYOCD	ENSG00000141052	NCI-H1048	Lung	-2	CCLE
MYOCD	ENSG00000141052	RERFLCKJ	Lung	-2	CCLE
MYOCD	ENSG00000141052	NCI-H2052	Lung/Pleura	-2	Crownbio
MYOCD	ENSG00000141052	CHAGOK1	Lung	-2	CCLE
MYOCD	ENSG00000141052	NCIH1618	Lung	-2	CCLE
MYOCD	ENSG00000141052	CORL24	Lung	-2	CCLE
MYOCD	ENSG00000141052	HCC4006	Lung	-2	CCLE
MYOCD	ENSG00000141052	Calu-3	Lung	-2	Crownbio
MYOCD	ENSG00000141052	NCI-H520	Lung	-2	CCLE
MYOCD	ENSG00000141052	H69AR	Lung	-2	Crownbio
MYOCD	ENSG00000141052	HCC-15	Lung	-2	CCLE
MYOCD	ENSG00000141052	DMS 114	Lung	-2	CCLE
MYOCD	ENSG00000141052	HCC366	Lung	-2	CCLE
MYOCD	ENSG00000141052	Calu-6	Lung	-2	CCLE
MYOCD	ENSG00000141052	DMS 114	Lung	-2	Crownbio
MYOCD	ENSG00000141052	ISTMES1	Lung/Pleura	-2	CCLE
MYOCD	ENSG00000141052	NCI-H2286	Lung	-2	CCLE
MYOCD	ENSG00000141052	SK-MES-1	Lung	-2	Crownbio
MYOCD	ENSG00000141052	NCIH1436	Lung	-2	CCLE
MYOCD	ENSG00000141052	RERFLCAI	Lung	-2	CCLE
MYOCD	ENSG00000141052	NCI-H727	Lung	-2	CCLE
MYOCD	ENSG00000141052	NCIH1930	Lung	-2	CCLE
MYOCD	ENSG00000141052	NCI-H1563	Lung	-2	Crownbio
MYOCD	ENSG00000141052	RERFLCSQ1	Lung	-2	CCLE
MYOCD	ENSG00000141052	NCI-H2170	Lung	-2	CCLE
MYOCD	ENSG00000141052	Hs 618.T	Lung	-1.8157	CCLE
MYOCD	ENSG00000141052	CORL88	Lung	-1.7936	CCLE
MYOCD	ENSG00000141052	MPP89	Lung/Pleura	-1.7752	CCLE
MYOCD	ENSG00000141052	LOU-NH91	Lung	-1.5422	CCLE
MYOCD	ENSG00000141052	NCI-H2126	Lung	-1.4774	CCLE
MYOCD	ENSG00000141052	MORCPR	Lung	-1.4614	CCLE
MYOCD	ENSG00000141052	NCI-H661	Lung	-1.3092	CCLE

MYOCD	ENSG00000141052	NCIH2405	Lung	-1.2953	CCLE
MYOCD	ENSG00000141052	LXF289	Lung	-1.2484	CCLE
MYOCD	ENSG00000141052	RERFLCMS	Lung	-1.1768	CCLE
MYOCD	ENSG00000141052	NCI-H23	Lung	-0.571	CCLE
MYOCD	ENSG00000141052	NCIH1623	Lung	-0.5646	CCLE
MYOCD	ENSG00000141052	NCI-H1915	Lung	-0.5468	CCLE
MYOCD	ENSG00000141052	NCIH2023	Lung	-0.5424	CCLE
MYOCD	ENSG00000141052	NCI-H1651	Lung	-0.5087	CCLE
MYOCD	ENSG00000141052	NCI-H1355	Lung	-0.2942	CCLE
MYOCD	ENSG00000141052	DM3	Lung/Pleura	0.0856	CCLE
MYOCD	ENSG00000141052	NCI-H1944	Lung	0.6655	CCLE
MYOCD	ENSG00000141052	HS229T	Lung	0.6761	CCLE
MYOCD	ENSG00000141052	HLFA	Lung	0.7521	CCLE
MYOCD	ENSG00000141052	DMS273	Lung	0.8674	CCLE
MYOCD	ENSG00000141052	NCI-H23	Lung	0.8939	Crownbio
MYOCD	ENSG00000141052	NCI-H2172	Lung	0.8988	CCLE
MYOCD	ENSG00000141052	A549	Lung	0.9331	Crownbio
MYOCD	ENSG00000141052	EPLC272H	Lung	1.174	CCLE
MYOCD	ENSG00000141052	RS5	Lung/Pleura	1.4677	CCLE
MYOCD	ENSG00000141052	NCIH2030	Lung	1.5998	CCLE
MYOCD	ENSG00000141052	EKVX	Lung	2.0817	CCLE
MYOCD	ENSG00000141052	A549	Lung	2.5044	CCLE
MYOCD	ENSG00000141052	NCI-H1651	Lung	2.5385	Crownbio

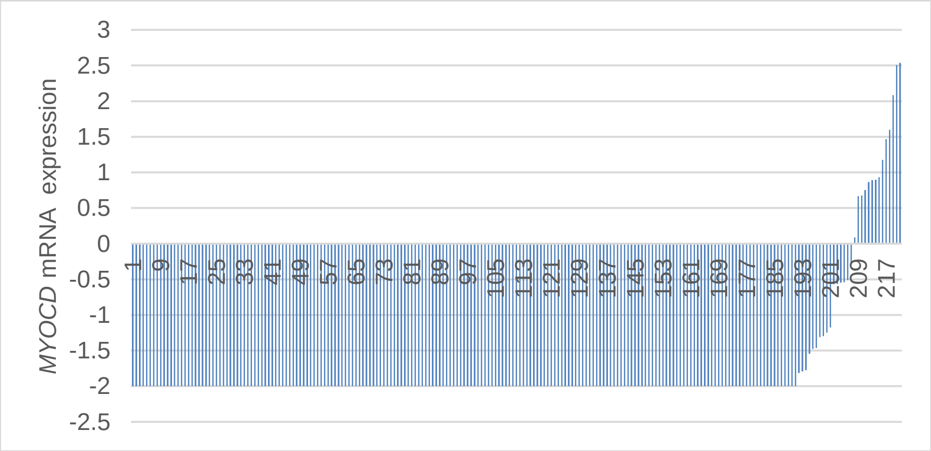

Table 2: IP-MS analysis of MYOCD associated protein partners.

Sample ID	Accession number	Protein description	Mascot score	MW	Matched queries	Matched peptides
GFP	IPI00743194	- Kappa light chain variable region (Fragment)	470	13174	36	2
	IPI00304925	HSPA1B;HSPA1A Heat shock 70 kDa protein 1A/1B	302	70009	14	10
	IPI00418471	VIM Vimentin	278	53619	13	13
	IPI00179330	RPS27A Ubiquitin-40S ribosomal protein S27a	256	17953	8	3
	IPI00003865	HSPA8 Isoform 1 of Heat shock cognate 71 kDa protein	238	70854	10	9
	IPI00829827	- 13 kDa protein	231	13071	25	4
	IPI00021439	ACTB Actin, cytoplasmic 1	195	41710	7	5
	IPI00453473	HIST2H4B;HIST1H4C;HIST1H4J;HIST1H4D;HIST1H4A;HIST2H4A;HIST1H4I;HIST1H4K;HIST1H	184	11360	8	3
	IPI00013296	RPS18;RPS18P9 40S ribosomal protein S18	177	17708	8	6
	IPI00645452	TUBB Uncharacterized protein	148	47736	8	6
	IPI00013881	HNRNPH1 Heterogeneous nuclear ribonucleoprotein H	142	49198	2	2
	IPI00020729	IRS4 Insulin receptor substrate 4	128	133685	2	1
	IPI00180675	TUBA1A Tubulin alpha-1A chain	127	50104	6	5
	IPI00007752	TUBB2C Tubulin beta-2C chain	124	49799	8	6
	IPI00022434	ALB Uncharacterized protein	118	71658	5	3
	IPI00376798	RPL11 Isoform 1 of 60S ribosomal protein L11	117	20240	2	1
	IPI00021263	YWHAZ 14-3-3 protein zeta/delta	114	27728	3	2
	IPI00018146	YWHAQ 14-3-3 protein theta	105	27747	2	2
	IPI00007188	SLC25A5 ADP/ATP translocase 2	104	32831	6	5
	IPI00007765	HSPA9 Stress-70 protein, mitochondrial	102	73635	2	2
	IPI00216049	HNRNPK Isoform 1 of Heterogeneous nuclear ribonucleoprotein K	101	50944	4	3
	IPI00000959	VIP Isoform 1 of VIP peptides	100	19157	4	1
	IPI00396378	HNRNPA2B1 Isoform B1 of Heterogeneous nuclear ribonucleoproteins A2/B1	96	37407	4	3
	IPI00449049	PARP1 Poly [ADP-ribose] polymerase 1	93	113012	4	4
	IPI00395474	SAMD1 Atherin	88	56018	1	1
	IPI00002214	KPNA2 Importin subunit alpha-2	85	57826	1	1
	IPI00003935	HIST2H2BE Histone H2B type 2-E	79	13912	8	5
	IPI00847768	DDX17 Uncharacterized protein	76	19189	1	1
	IPI00216592	HNRNPC Isoform C1 of Heterogeneous nuclear ribonucleoproteins C1/C2	75	32318	3	2
	IPI00410693	SERBP1 SERPINE1 mRNA binding protein 1, isoform CRA_d	75	50890	2	2
	IPI00651653	DDX17 probable ATP-dependent RNA helicase DDX17 isoform 3	72	80407	2	2
	IPI00012493	SNORD54;RPS20 40S ribosomal protein S20	70	13364	2	2
	IPI00009379	IGHMBP2 DNA-binding protein SMUBP-2	67	109082	2	1
	IPI00157561	TAB2 Isoform 2 of TGF-beta-activated kinase 1 and MAP3K7-binding protein 2	65	58095	1	1
	IPI00171611	HIST2H3D;HIST2H3A;HIST2H3C Histone H3.2	63	15379	5	4
	IPI00305289	KIF11 Kinesin-like protein KIF11	61	119085	6	6
	IPI00788837	PCBP2 cDNA FLJ54752, highly similar to Poly(rC)-binding protein 2	61	29718	1	1
	IPI00012066	PCBP2 poly(rC)-binding protein 2 isoform b	60	38197	2	2
	IPI00032314	LAGE3 L antigen family member 3	59	14795	1	1
	IPI00218993	HSPH1 Isoform Beta of Heat shock protein 105 kDa	58	92057	2	2
	IPI00304692	RBMX Heterogeneous nuclear ribonucleoprotein G	58	42306	2	2
	IPI00171903	HNRNPM Isoform 1 of Heterogeneous nuclear ribonucleoprotein M	58	77464	2	2
	IPI00095891	GNAS Isoform XLas-1 of Guanine nucleotide-binding protein G(s) subunit alpha i	57	110956	1	1
	IPI00044665	FAM117B Isoform 2 of Protein FAM117B	56	29410	1	1
	IPI00106509	HNRNPAB Isoform 4 of Heterogeneous nuclear ribonucleoprotein A/B	54	30682	1	1
	IPI00027107	TUFM elongation factor Tu, mitochondrial precursor	53	49843	1	1
	IPI00939612	SLC22A20 Similar to Solute carrier family 22, member 20	53	13446	6	1
	IPI00438229	TRIM28 Isoform 1 of Transcription intermediary factor 1-beta	52	88493	1	1
	IPI00301434	BOLA2B;BOLA2 BolA-like protein 2	49	16922	1	1
	IPI00217465	HIST1H1C Histone H1.2	48	21352	3	3
	IPI00215965	HNRNPA1 Isoform A1-B of Heterogeneous nuclear ribonucleoprotein A1	47	38723	2	2
	IPI00009542	MAGED2 Isoform 1 of Melanoma-associated antigen D2	46	64914	1	1
	IPI00221089	RPS13 40S ribosomal protein S13	45	17212	1	1
	IPI00014424	EEF1A2 Elongation factor 1-alpha 2	42	50438	1	1
	IPI00024933	RPL12 Isoform 1 of 60S ribosomal protein L12	41	17808	2	2
	IPI00011253	RPS3 40S ribosomal protein S3	41	26671	3	2
	IPI00216870	IL16 Uncharacterized protein	40	48362	1	1
	IPI00012772	RPL8 60S ribosomal protein L8	40	28007	1	1
	IPI00022228	HDLBP Vigilin	40	141368	1	1
	IPI00470559	KANK2 Isoform 1 of KN motif and ankyrin repeat domain-containing protein 2	38	91118	1	1
	IPI00915941	HSPD1 Uncharacterized protein	38	25210	1	1
	IPI00306332	RPL24 60S ribosomal protein L24	37	17768	1	1
	IPI00290791	CASP10 Isoform A of Caspase-10	36	58913	1	1
	IPI00022432	TTR Transthyretin	35	15877	1	1
	IPI00291510	IMPDH2 Inosine-5˜-monophosphate dehydrogenase 2	35	55770	1	1
	IPI00465439	ALDOA Fructose-bisphosphate aldolase A	34	39395	1	1
	IPI00784154	HSPD1 60 kDa heat shock protein, mitochondrial	34	61016	2	2
	IPI00871311	ZYX cDNA FLJ53160, highly similar to Zyxin	34	51995	2	2
	IPI00032003	EMD Emerin	34	28976	2	2
	IPI00217030	RPS4X 40S ribosomal protein S4, X isoform	33	29579	1	1
	IPI00930144	H2AFJ Histone H2A	33	12114	1	1
	IPI01025580	- Possible J 56 gene segment (Fragment)	33	2212	5	1
	IPI00018262	ANP32C Acidic leucine-rich nuclear phosphoprotein 32 family member C	33	26746	1	1
	IPI00000001	STAU1 Isoform Long of Double-stranded RNA-binding protein Staufen homolog 1	33	63143	1	1
	IPI00220740	NPM1 Isoform 2 of Nucleophosmin	33	29446	1	1
	IPI00045109	HIST1H2AA Histone H2A type 1-A	32	14225	2	2
	IPI00013485	RPS2 40S ribosomal protein S2	32	31305	1	1

	IPI00215637	DDX3X ATP-dependent RNA helicase DDX3X	30	73198	2	2
	IPI00031820	FARSA Phenylalanyl-tRNA synthetase alpha chain	30	57528	1	1
	IPI00023048	EEF1D Isoform 1 of Elongation factor 1-delta	30	31103	1	1
	IPI00004065	ART4 Ecto-ADP-ribosyltransferase 4	30	35854	1	1
	IPI00009631	ST3GAL4 Isoform 1 of CMP-N-acetylneuraminate-beta-galactosamide-alpha-2,3-sial	30	38021	1	1
	IPI00102918	ATRIP Isoform 2 of ATR-interacting protein	29	82951	1	1
	IPI00217541	DDX51 ATP-dependent RNA helicase DDX51	29	72413	2	1
	IPI00030131	TMPO Isoform Beta of Lamina-associated polypeptide 2, isoforms beta/gamma	28	50639	1	1
	IPI00015180	SHROOM2 Protein Shroom2	28	176303	1	1
	IPI00021840	RPS6 40S ribosomal protein S6	27	28663	1	1
	IPI00008529	RPLP2 60S acidic ribosomal protein P2	27	11658	2	1
	IPI00000643	BAG2 BAG family molecular chaperone regulator 2	27	23757	1	1
	IPI00008530	RPLP0 60S acidic ribosomal protein P0	27	34252	1	1
	IPI00011200	PHGDH D-3-phosphoglycerate dehydrogenase	26	56614	1	1
	IPI00414860	RPL37A 60S ribosomal protein L37a	26	10268	1	1
	IPI00022694	PSMD4 Isoform Rpn10A of 26S proteasome non-ATPase regulatory subunit 4	25	40711	1	1
	IPI00012750	RPS25 40S ribosomal protein S25	25	13734	2	2
	IPI00217985	DOCK3 Dedicator of cytokinesis protein 3	25	232954	1	1
	IPI00064765	RPL10L 60S ribosomal protein L10-like	23	24503	1	1
	IPI01012677	KIAA1211 cDNA FLJ60370, weakly similar to Homo sapiens retinitis pigmentosa G1	23	97460	1	1
	IPI00172636	CAMK2D Isoform Delta 6 of Calcium/calmodulin-dependent protein kinase type II	22	54093	1	1
	IPI00186290	EEF2 Elongation factor 2	22	95277	1	1
	IPI00012535	DNAJA1 DnaJ homolog subfamily A member 1	22	44839	1	1
	IPI00007935	PDLIM5 Isoform 1 of PDZ and LIM domain protein 5	22	63904	1	1
	IPI00021266	RPL23A;SNORD4A 60S ribosomal protein L23a	21	17684	1	1
	IPI00896367	TBXA2R Uncharacterized protein	21	26655	1	1
	IPI00064219	NEURL4 Isoform 1 of Neuralized-like protein 4	20	166802	1	1
	IPI00382470	HSP90AA1 Isoform 2 of Heat shock protein HSP 90-alpha	20	98099	1	1
	IPI00896542	SPRED2 sprouty-related, EVH1 domain-containing protein 2 isoform b	19	47002	1	1
	IPI00289799	ZMIZ2 Isoform 2 of Zinc finger MIZ domain-containing protein 2	19	93922	1	1
	IPI00300631	SAFB Scaffold attachment factor B1	18	102580	1	1
	IPI00216813	ATXN7L2 Ataxin-7-like protein 2	18	77132	1	1
	IPI00981973	PKNOX1 Uncharacterized protein	18	34498	1	1
	IPI00025039	FBL rRNA 2~-O-methyltransferase fibrillarin	17	33763	1	1
	IPI00479058	RPS15 40S ribosomal protein S15	17	17029	1	1
	IPI00005969	CAPZA1 F-actin-capping protein subunit alpha-1	17	32902	1	1
	IPI00008708	RSL1D1 Ribosomal L1 domain-containing protein 1	16	54939	1	1
	IPI00386170	SSFA2 Isoform 3 of Sperm-specific antigen 2	15	137773	1	1
	IPI00065313	TAF8 Isoform 2 of Transcription initiation factor TFIID subunit 8	14	34414	1	1
MYOCD	IPI00743194	- Kappa light chain variable region (Fragment)	580	13174	34	2
	IPI00305289	KIF11 Kinesin-like protein KIF11	523	119085	32	27
	IPI00021439	ACTB Actin, cytoplasmic 1	381	41710	15	8
	IPI00829827	- 13 kDa protein	296	13071	29	3
	IPI00031812	YBX1 Nuclease-sensitive element-binding protein 1	225	35903	6	4
	IPI00163729	MKL1 MKL1 protein	206	84994	11	10
	IPI00304925	HSPA1B;HSPA1A Heat shock 70 kDa protein 1A/1B	203	70009	13	10
	IPI00013296	RPS18;RPS18P9 40S ribosomal protein S18	176	17708	8	7
	IPI00218055	MYOCD Isoform 1 of Myocardin	171	101934	11	9
	IPI00064328	PRMT5 protein arginine N-methyltransferase 5 isoform b	165	71275	9	7
	IPI00026612	PPM1B Isoform Beta-1 of Protein phosphatase 1B	163	52609	5	4
	IPI00012079	EIF4B Eukaryotic translation initiation factor 4B	148	69110	6	6
	IPI00003865	HSPA8 Isoform 1 of Heat shock cognate 71 kDa protein	146	70854	8	6
	IPI00217465	HIST1H1C Histone H1.2	125	21352	5	5
	IPI00022434	ALB Uncharacterized protein	106	71658	6	2
	IPI00013881	HNRNPH1 Heterogeneous nuclear ribonucleoprotein H	105	49198	2	1
	IPI00376798	RPL11 Isoform 1 of 60S ribosomal protein L11	99	20240	2	1
	IPI00179709	TUBA3D;TUBA3C Isoform 1 of Tubulin alpha-3C/D chain	99	49928	5	4
	IPI00183526	NCL Uncharacterized protein	95	50920	5	5
	IPI00410693	SERBP1 SERPINE1 mRNA binding protein 1, isoform CRA_d	90	50890	2	2
	IPI00012202	WDR77 Methylosome protein 50	86	36701	3	3
	IPI00003881	HNRNPF Heterogeneous nuclear ribonucleoprotein F	81	45643	1	1
	IPI00031801	CSDA Isoform 1 of DNA-binding protein A	77	40066	1	1
	IPI00106955	C11orf84 Uncharacterized protein C11orf84	77	41011	3	2
	IPI00021263	YWHAZ 14-3-3 protein zeta/delta	65	27728	2	2
	IPI00009379	IGHMBP2 DNA-binding protein SMUBP-2	64	109082	3	1
	IPI00410590	LSM14A Isoform 2 of Protein LSM14 homolog A	62	50523	3	3
	IPI00301434	BOLA2B;BOLA2 BolA-like protein 2	61	16922	1	1
	IPI00012493	SNORD54;RPS20 40S ribosomal protein S20	60	13364	1	1
	IPI00004511	PFKFB3 Isoform 1 of 6-phosphofructo-2-kinase/fructose-2,6-biphosphatase 3	59	59571	1	1
	IPI00008868	MAP1B Microtubule-associated protein 1B	57	270468	2	1
	IPI00465361	RPL13 60S ribosomal protein L13	57	24247	1	1
	IPI00003362	HSPA5 78 kDa glucose-regulated protein	56	72288	3	3
	IPI00007765	HSPA9 Stress-70 protein, mitochondrial	56	73635	1	1
	IPI00007144	RPL26L1 60S ribosomal protein L26-like 1	56	17246	1	1
	IPI00012772	RPL8 60S ribosomal protein L8	56	28007	5	4
	IPI00003935	HIST2H2BE Histone H2B type 2-E	56	13912	3	2
	IPI00790342	RPL6 60S ribosomal protein L6	54	32871	2	2
	IPI00290791	CASP10 Isoform A of Caspase-10	52	58913	4	1
	IPI00027729	CSNK1E Casein kinase I isoform epsilon	51	47285	1	1
	IPI00007188	SLC25A5 ADP/ATP translocase 2	51	32831	2	2
	IPI00383808	- Ig Kappa chain V-IV region STH (Fragment)	49	12053	1	1

IPI00215719	RPL18 60S ribosomal protein L18	48	21621	1	1
IPI00375731	RBM10 Isoform 1 of RNA-binding protein 10	47	103469	2	2
IPI00008530	RPLP0 60S acidic ribosomal protein P0	47	34252	2	1
IPI00247583	RPL21;SNORD102;SNORA27;RPL21P19 60S ribosomal protein L21	47	18553	1	1
IPI00784154	HSPD1 60 kDa heat shock protein, mitochondrial	46	61016	1	1
IPI00022694	PSMD4 Isoform Rpn10A of 26S proteasome non-ATPase regulatory subunit 4	45	40711	2	2
IPI01020995	SCYL2 Uncharacterized protein	45	77004	2	2
IPI00004506	KCTD5 BTB/POZ domain-containing protein KCTD5	45	26076	1	1
IPI00396218	SCYL2 SCY1-like protein 2	44	103642	3	3
IPI00026089	SF3B1 Splicing factor 3B subunit 1	44	145738	1	1
IPI00455348	KCTD2 BTB/POZ domain-containing protein KCTD2	44	28509	1	1
IPI00220528	SNRPF Small nuclear ribonucleoprotein F	44	9719	1	1
IPI00220740	NPM1 Isoform 2 of Nucleophosmin	43	29446	1	1
IPI00215637	DDX3X ATP-dependent RNA helicase DDX3X	41	73198	1	1
IPI00216592	HNRNPC Isoform C1 of Heterogeneous nuclear ribonucleoproteins C1/C2	41	32318	1	1
IPI00221106	SF3B2 Splicing factor 3B subunit 2	41	100165	2	2
IPI00019580	PLG Plasminogen	41	90510	1	1
IPI00045109	HIST1H2AA Histone H2A type 1-A	39	14225	1	1
IPI00554723	SNORA70;RPL10 60S ribosomal protein L10	38	24588	2	2
IPI00418471	VIM Vimentin	38	53619	3	3
IPI00219729	SLC25A11 Mitochondrial 2-oxoglutarate/malate carrier protein	37	34040	1	1
IPI00215780	RPS19 40S ribosomal protein S19	37	16051	1	1
IPI00161600	C17orf79 Cooperator of PRMT5	37	20054	2	1
IPI00002127	DNAH1 Isoform 2 of Dynein heavy chain 1, axonemal	37	485611	1	1
IPI00011253	RPS3 40S ribosomal protein S3	36	26671	2	2
IPI00015963	TRPA1 Transient receptor potential cation channel subfamily A member 1	35	127418	1	1
IPI00470559	KANK2 Isoform 1 of KN motif and ankyrin repeat domain-containing protein 2	34	91118	1	1
IPI00022334	OAT Ornithine aminotransferase, mitochondrial	34	48504	1	1
IPI00008575	KHDRBS1 Isoform 1 of KH domain-containing, RNA-binding, signal transduction-as	34	48197	1	1
IPI01022997	- Uncharacterized protein	33	12936	1	1
IPI00029731	RPL35A 60S ribosomal protein L35a	33	12530	1	1
IPI00026271	RPS14 40S ribosomal protein S14	33	16263	1	1
IPI00000877	HYOU1 Hypoxia up-regulated protein 1	33	111266	1	1
IPI00219155	RPL27 60S ribosomal protein L27	32	15788	1	1
IPI00004065	ART4 Ecto-ADP-ribosyltransferase 4	32	35854	1	1
IPI00008524	PABPC1 Isoform 1 of Polyadenylate-binding protein 1	32	70626	1	1
IPI00299573	RPL7A;SNORD24 60S ribosomal protein L7a	32	29977	1	1
IPI00027235	ATRN Isoform 1 of Attractin	31	158432	1	1
IPI00044583	TRERF1 Isoform 1 of Transcriptional-regulating factor 1	31	132173	1	1
IPI00412579	RPL10A 60S ribosomal protein L10a	31	24816	1	1
IPI00018971	TRIM21 Isoform 1 of E3 ubiquitin-protein ligase TRIM21	31	54135	2	2
IPI00000001	STAU1 Isoform Long of Double-stranded RNA-binding protein Staufen homolog 1	31	63143	1	1
IPI00645452	TUBB Uncharacterized protein	31	47736	2	2
IPI00179964	PTBP1 Isoform 1 of Polypyrimidine tract-binding protein 1	30	57186	1	1
IPI00171903	HNRNPM Isoform 1 of Heterogeneous nuclear ribonucleoprotein M	30	77464	3	3
IPI00304612	SNORD32A;RPL13A;SNORD33;SNORD34 60S ribosomal protein L13a	29	23562	1	1
IPI00030131	TMPO Isoform Beta of Lamina-associated polypeptide 2, isoforms beta/gamma	28	50639	1	1
IPI00003406	DBN1 Isoform 1 of Drebrin	28	71385	1	1
IPI00218064	CLECL1 C-type lectin-like domain family 1	28	19103	1	1
IPI00025329	RPL19 60S ribosomal protein L19	27	23451	1	1
IPI00335641	MKL2 cDNA FLJ61243, highly similar to MKL/myocardin-like protein 2	27	119092	2	2
IPI01025580	- Possible J 56 gene segment (Fragment)	27	2212	2	1
IPI00221089	RPS13 40S ribosomal protein S13	26	17212	1	1
IPI00306332	RPL24 60S ribosomal protein L24	26	17768	1	1
IPI00759479	IQSEC2 Isoform 1 of IQ motif and SEC7 domain-containing protein 2	26	161637	1	1
IPI00217030	RPS4X 40S ribosomal protein S4, X isoform	25	29579	1	1
IPI00006079	BCLAF1 Isoform 1 of Bcl-2-associated transcription factor 1	25	106059	1	1
IPI00651653	DDX17 probable ATP-dependent RNA helicase DDX17 isoform 3	24	80407	1	1
IPI00964841	APBB2 Protein	23	4656	1	1
IPI00000959	VIP Isoform 1 of VIP peptides	23	19157	1	1
IPI00217541	DDX51 ATP-dependent RNA helicase DDX51	22	72413	1	1
IPI00026328	TXNDC12 Thioredoxin domain-containing protein 12	21	19194	1	1
IPI00024933	RPL12 Isoform 1 of 60S ribosomal protein L12	20	17808	1	1
IPI00735705	LOC100128402 Similar to U1 small nuclear ribonucleoprotein C	19	7380	1	1
IPI00021924	H1FX Histone H1x	19	22474	1	1
IPI00010153	RPL23 60S ribosomal protein L23	18	14856	1	1
IPI00943803	KIAA0913 Protein	17	138703	1	1
IPI00289499	ATIC Bifunctional purine biosynthesis protein PURH	17	64575	1	1
IPI00186290	EEF2 Elongation factor 2	17	95277	1	1
IPI00385236	NTN4 Isoform 3 of Netrin-4	17	66308	1	1
IPI00163718	VPS54 Isoform 4 of Vacuolar protein sorting-associated protein 54	16	109292	1	1
IPI00000875	EEF1G cDNA FLJ56389, highly similar to Elongation factor 1-gamma	15	56114	1	1
IPI00171117	COMMD1 COMM domain-containing protein 1	15	21165	1	1
IPI00008708	RSL1D1 Ribosomal L1 domain-containing protein 1	14	54939	1	1
IPI00796239	TNFRSF10B Uncharacterized protein	13	9382	1	1

Table 3: The clinical database is origin from UCSC Xena (http://xena.ucsc.edu/compare-tissue/). MYOCD expression was analyzed, the median value was chosen as a cutoff. Then analysis the percentage patients with low expression of MYOCD in patients with KRAS mutations.

sample	_study	_sample_type	_primary_ site	primary disease or tissue	MYOCD expression	KRAS mutation
TCGA-44-6146-01	TCGA	Primary Tumor	Lung	Lung Adenocarcinoma	10.87	KRAS
TCGA-55-8207-01	TCGA	Primary Tumor	Lung	Lung Adenocarcinoma	9.28	KRAS
TCGA-55-8097-01	TCGA	Primary Tumor	Lung	Lung Adenocarcinoma	9.271	KRAS
TCGA-LA-A7SW-01	TCGA	Primary Tumor	Lung	Lung Squamous Cell Carcinom	8.944	KRAS
TCGA-73-7498-01	TCGA	Primary Tumor	Lung	Lung Adenocarcinoma	8.691	KRAS
TCGA-97-8179-01	TCGA	Primary Tumor	Lung	Lung Adenocarcinoma	8.503	KRAS
TCGA-78-7160-01	TCGA	Primary Tumor	Lung	Lung Adenocarcinoma	8.1	KRAS
TCGA-97-A4M5-01	TCGA	Primary Tumor	Lung	Lung Adenocarcinoma	7.756	KRAS
TCGA-NJ-A55R-01	TCGA	Primary Tumor	Lung	Lung Adenocarcinoma	7.708	KRAS
TCGA-MP-A4TD-01	TCGA	Primary Tumor	Lung	Lung Adenocarcinoma	7.692	KRAS
TCGA-55-8512-01	TCGA	Primary Tumor	Lung	Lung Adenocarcinoma	7.659	KRAS
TCGA-MP-A4TI-01	TCGA	Primary Tumor	Lung	Lung Adenocarcinoma	7.42	KRAS
TCGA-55-7284-01	TCGA	Primary Tumor	Lung	Lung Adenocarcinoma	7.416	KRAS
TCGA-97-7941-01	TCGA	Primary Tumor	Lung	Lung Adenocarcinoma	7.374	KRAS
TCGA-55-7728-01	TCGA	Primary Tumor	Lung	Lung Adenocarcinoma	7.345	KRAS
TCGA-44-7671-01	TCGA	Primary Tumor	Lung	Lung Adenocarcinoma	7.266	KRAS
TCGA-97-7938-01	TCGA	Primary Tumor	Lung	Lung Adenocarcinoma	7.251	KRAS
TCGA-93-8067-01	TCGA	Primary Tumor	Lung	Lung Adenocarcinoma	7.161	KRAS
TCGA-49-AARO-01	TCGA	Primary Tumor	Lung	Lung Adenocarcinoma	7.123	KRAS
TCGA-95-A4VP-01	TCGA	Primary Tumor	Lung	Lung Adenocarcinoma	7.116	KRAS
TCGA-50-8459-01	TCGA	Primary Tumor	Lung	Lung Adenocarcinoma	6.99	KRAS
TCGA-49-4505-01	TCGA	Primary Tumor	Lung	Lung Adenocarcinoma	6.968	KRAS
TCGA-55-7283-01	TCGA	Primary Tumor	Lung	Lung Adenocarcinoma	6.955	KRAS
TCGA-75-7030-01	TCGA	Primary Tumor	Lung	Lung Adenocarcinoma	6.931	KRAS
TCGA-55-6975-01	TCGA	Primary Tumor	Lung	Lung Adenocarcinoma	6.923	KRAS
TCGA-55-1595-01	TCGA	Primary Tumor	Lung	Lung Adenocarcinoma	6.92	KRAS
TCGA-35-3615-01	TCGA	Primary Tumor	Lung	Lung Adenocarcinoma	6.9	KRAS
TCGA-05-4417-01	TCGA	Primary Tumor	Lung	Lung Adenocarcinoma	6.85	KRAS
TCGA-69-8253-01	TCGA	Primary Tumor	Lung	Lung Adenocarcinoma	6.799	KRAS
TCGA-75-7027-01	TCGA	Primary Tumor	Lung	Lung Adenocarcinoma	6.75	KRAS
TCGA-69-8254-01	TCGA	Primary Tumor	Lung	Lung Adenocarcinoma	6.709	KRAS
TCGA-L9-A443-01	TCGA	Primary Tumor	Lung	Lung Adenocarcinoma	6.678	KRAS
TCGA-62-A46S-01	TCGA	Primary Tumor	Lung	Lung Adenocarcinoma	6.637	KRAS

TCGA-78-7540-01	TCGA	Primary Tumor	Lung	Lung Adenocarcinoma	6.626	KRAS	
TCGA-05-4433-01	TCGA	Primary Tumor	Lung	Lung Adenocarcinoma	6.541	KRAS	
TCGA-55-6983-01	TCGA	Primary Tumor	Lung	Lung Adenocarcinoma	6.498	KRAS	
TCGA-MP-A4TK-01	TCGA	Primary Tumor	Lung	Lung Adenocarcinoma	6.446	KRAS	
TCGA-93-7347-01	TCGA	Primary Tumor	Lung	Lung Adenocarcinoma	6.413	KRAS	
TCGA-49-4510-01	TCGA	Primary Tumor	Lung	Lung Adenocarcinoma	6.385	KRAS	
TCGA-95-A4VK-01	TCGA	Primary Tumor	Lung	Lung Adenocarcinoma	6.348	KRAS	
TCGA-69-7980-01	TCGA	Primary Tumor	Lung	Lung Adenocarcinoma	6.297	KRAS	
TCGA-67-3774-01	TCGA	Primary Tumor	Lung	Lung Adenocarcinoma	6.296	KRAS	
TCGA-78-7167-01	TCGA	Primary Tumor	Lung	Lung Adenocarcinoma	6.266	KRAS	
TCGA-44-7661-01	TCGA	Primary Tumor	Lung	Lung Adenocarcinoma	6.265	KRAS	
TCGA-86-8359-01	TCGA	Primary Tumor	Lung	Lung Adenocarcinoma	6.25	KRAS	
TCGA-99-8025-01	TCGA	Primary Tumor	Lung	Lung Adenocarcinoma	6.239	KRAS	
TCGA-67-4679-01	TCGA	Primary Tumor	Lung	Lung Adenocarcinoma	6.218	KRAS	
TCGA-78-7145-01	TCGA	Primary Tumor	Lung	Lung Adenocarcinoma	6.208	KRAS	
TCGA-69-7978-01	TCGA	Primary Tumor	Lung	Lung Adenocarcinoma	6.136	KRAS	
TCGA-44-6776-01	TCGA	Primary Tumor	Lung	Lung Adenocarcinoma	6.073	KRAS	
TCGA-78-7161-01	TCGA	Primary Tumor	Lung	Lung Adenocarcinoma	6.069	KRAS	
TCGA-55-8508-01	TCGA	Primary Tumor	Lung	Lung Adenocarcinoma	6.065	KRAS	
TCGA-55-8090-01	TCGA	Primary Tumor	Lung	Lung Adenocarcinoma	6.049	KRAS	MYOCD high expression
TCGA-78-7166-01	TCGA	Primary Tumor	Lung	Lung Adenocarcinoma	6.023	KRAS	
TCGA-75-6206-01	TCGA	Primary Tumor	Lung	Lung Adenocarcinoma	6.014	KRAS	
TCGA-86-A456-01	TCGA	Primary Tumor	Lung	Lung Adenocarcinoma	5.999	KRAS	
TCGA-95-7039-01	TCGA	Primary Tumor	Lung	Lung Adenocarcinoma	5.976	KRAS	
TCGA-97-7554-01	TCGA	Primary Tumor	Lung	Lung Adenocarcinoma	5.966	KRAS	
TCGA-MP-A4SY-01	TCGA	Primary Tumor	Lung	Lung Adenocarcinoma	5.965	KRAS	
TCGA-55-7281-01	TCGA	Primary Tumor	Lung	Lung Adenocarcinoma	5.845	KRAS	
TCGA-44-6145-01	TCGA	Primary Tumor	Lung	Lung Adenocarcinoma	5.837	KRAS	
TCGA-J2-A4AG-01	TCGA	Primary Tumor	Lung	Lung Adenocarcinoma	5.805	KRAS	
TCGA-69-7974-01	TCGA	Primary Tumor	Lung	Lung Adenocarcinoma	5.735	KRAS	
TCGA-50-5051-01	TCGA	Primary Tumor	Lung	Lung Adenocarcinoma	5.73	KRAS	
TCGA-55-7576-01	TCGA	Primary Tumor	Lung	Lung Adenocarcinoma	5.674	KRAS	
TCGA-55-7907-01	TCGA	Primary Tumor	Lung	Lung Adenocarcinoma	5.661	KRAS	
TCGA-NJ-A55O-01	TCGA	Primary Tumor	Lung	Lung Adenocarcinoma	5.583	KRAS	
TCGA-93-A4JO-01	TCGA	Primary Tumor	Lung	Lung Adenocarcinoma	5.564	KRAS	
TCGA-55-7910-01	TCGA	Primary Tumor	Lung	Lung Adenocarcinoma	5.542	KRAS	
TCGA-99-8032-01	TCGA	Primary Tumor	Lung	Lung Adenocarcinoma	5.54	KRAS	
TCGA-55-8299-01	TCGA	Primary Tumor	Lung	Lung Adenocarcinoma	5.478	KRAS	
TCGA-99-8028-01	TCGA	Primary Tumor	Lung	Lung Adenocarcinoma	5.47	KRAS	

TCGA-50-5932-01	TCGA	Primary Tumor	Lung	Lung Adenocarcinoma	5.468	KRAS
TCGA-21-1078-01	TCGA	Primary Tumor	Lung	Lung Squamous Cell Carcinom	5.429	KRAS
TCGA-86-8076-01	TCGA	Primary Tumor	Lung	Lung Adenocarcinoma	5.427	KRAS
TCGA-55-6970-01	TCGA	Primary Tumor	Lung	Lung Adenocarcinoma	5.363	KRAS
TCGA-44-8117-01	TCGA	Primary Tumor	Lung	Lung Adenocarcinoma	5.357	KRAS
TCGA-55-8203-01	TCGA	Primary Tumor	Lung	Lung Adenocarcinoma	5.334	KRAS
TCGA-55-6642-01	TCGA	Primary Tumor	Lung	Lung Adenocarcinoma	5.274	KRAS
TCGA-MP-A4T4-01	TCGA	Primary Tumor	Lung	Lung Adenocarcinoma	5.251	KRAS
TCGA-97-8176-01	TCGA	Primary Tumor	Lung	Lung Adenocarcinoma	5.22	KRAS
TCGA-44-7672-01	TCGA	Primary Tumor	Lung	Lung Adenocarcinoma	5.174	KRAS
TCGA-80-5608-01	TCGA	Primary Tumor	Lung	Lung Adenocarcinoma	5.122	KRAS
TCGA-73-4662-01	TCGA	Primary Tumor	Lung	Lung Adenocarcinoma	5.104	KRAS
TCGA-63-6202-01	TCGA	Primary Tumor	Lung	Lung Squamous Cell Carcinom	5.076	KRAS
TCGA-91-6828-01	TCGA	Primary Tumor	Lung	Lung Adenocarcinoma	5.065	KRAS
TCGA-91-6849-01	TCGA	Primary Tumor	Lung	Lung Adenocarcinoma	5.041	KRAS
TCGA-55-8514-01	TCGA	Primary Tumor	Lung	Lung Adenocarcinoma	5.04	KRAS
TCGA-05-4415-01	TCGA	Primary Tumor	Lung	Lung Adenocarcinoma	5.03	KRAS
TCGA-50-5941-01	TCGA	Primary Tumor	Lung	Lung Adenocarcinoma	5.004	KRAS
TCGA-50-5936-01	TCGA	Primary Tumor	Lung	Lung Adenocarcinoma	4.973	KRAS
TCGA-55-6984-01	TCGA	Primary Tumor	Lung	Lung Adenocarcinoma	4.953	KRAS
TCGA-97-A4M0-01	TCGA	Primary Tumor	Lung	Lung Adenocarcinoma	4.932	KRAS
TCGA-05-4244-01	TCGA	Primary Tumor	Lung	Lung Adenocarcinoma	4.926	KRAS
TCGA-75-5126-01	TCGA	Primary Tumor	Lung	Lung Adenocarcinoma	4.897	KRAS
TCGA-44-A47A-01	TCGA	Primary Tumor	Lung	Lung Adenocarcinoma	4.893	KRAS
TCGA-83-5908-01	TCGA	Primary Tumor	Lung	Lung Adenocarcinoma	4.78	KRAS
TCGA-78-7148-01	TCGA	Primary Tumor	Lung	Lung Adenocarcinoma	4.756	KRAS
TCGA-55-7726-01	TCGA	Primary Tumor	Lung	Lung Adenocarcinoma	4.744	KRAS
TCGA-J2-8194-01	TCGA	Primary Tumor	Lung	Lung Adenocarcinoma	4.662	KRAS
TCGA-NJ-A4YG-01	TCGA	Primary Tumor	Lung	Lung Adenocarcinoma	4.656	KRAS
TCGA-99-8033-01	TCGA	Primary Tumor	Lung	Lung Adenocarcinoma	4.643	KRAS
TCGA-MN-A4N5-01	TCGA	Primary Tumor	Lung	Lung Adenocarcinoma	4.515	KRAS
TCGA-86-7953-01	TCGA	Primary Tumor	Lung	Lung Adenocarcinoma	4.481	KRAS
TCGA-55-8616-01	TCGA	Primary Tumor	Lung	Lung Adenocarcinoma	4.462	KRAS
TCGA-95-A4VN-01	TCGA	Primary Tumor	Lung	Lung Adenocarcinoma	4.434	KRAS
TCGA-44-3917-01	TCGA	Primary Tumor	Lung	Lung Adenocarcinoma	4.407	KRAS
TCGA-62-8398-01	TCGA	Primary Tumor	Lung	Lung Adenocarcinoma	4.407	KRAS
TCGA-62-A46R-01	TCGA	Primary Tumor	Lung	Lung Adenocarcinoma	4.381	KRAS
TCGA-55-7725-01	TCGA	Primary Tumor	Lung	Lung Adenocarcinoma	4.358	KRAS
TCGA-73-4670-01	TCGA	Primary Tumor	Lung	Lung Adenocarcinoma	4.327	KRAS

TCGA-55-8302-01	TCGA	Primary Tumor	Lung	Lung Adenocarcinoma	4.317	KRAS	
TCGA-69-7973-01	TCGA	Primary Tumor	Lung	Lung Adenocarcinoma	4.317	KRAS	
TCGA-86-A4JF-01	TCGA	Primary Tumor	Lung	Lung Adenocarcinoma	4.286	KRAS	
TCGA-MP-A4T7-01	TCGA	Primary Tumor	Lung	Lung Adenocarcinoma	4.284	KRAS	
TCGA-73-4677-01	TCGA	Primary Tumor	Lung	Lung Adenocarcinoma	4.233	KRAS	
TCGA-78-8655-01	TCGA	Primary Tumor	Lung	Lung Adenocarcinoma	4.138	KRAS	
TCGA-78-7539-01	TCGA	Primary Tumor	Lung	Lung Adenocarcinoma	4.108	KRAS	
TCGA-MP-A4T8-01	TCGA	Primary Tumor	Lung	Lung Adenocarcinoma	4.028	KRAS	
TCGA-64-5775-01	TCGA	Primary Tumor	Lung	Lung Adenocarcinoma	4.014	KRAS	
TCGA-05-4427-01	TCGA	Primary Tumor	Lung	Lung Adenocarcinoma	3.999	KRAS	
TCGA-05-4250-01	TCGA	Primary Tumor	Lung	Lung Adenocarcinoma	3.973	KRAS	
TCGA-86-8674-01	TCGA	Primary Tumor	Lung	Lung Adenocarcinoma	3.903	KRAS	
TCGA-67-3773-01	TCGA	Primary Tumor	Lung	Lung Adenocarcinoma	3.897	KRAS	
TCGA-55-7914-01	TCGA	Primary Tumor	Lung	Lung Adenocarcinoma	3.876	KRAS	
TCGA-05-4418-01	TCGA	Primary Tumor	Lung	Lung Adenocarcinoma	3.691	KRAS	
TCGA-MP-A4TF-01	TCGA	Primary Tumor	Lung	Lung Adenocarcinoma	3.583	KRAS	
TCGA-86-7713-01	TCGA	Primary Tumor	Lung	Lung Adenocarcinoma	3.526	KRAS	
TCGA-64-5815-01	TCGA	Primary Tumor	Lung	Lung Adenocarcinoma	3.509	KRAS	
TCGA-66-2769-01	TCGA	Primary Tumor	Lung	Lung Squamous Cell Carcinom	3.496	KRAS	
TCGA-21-5783-01	TCGA	Primary Tumor	Lung	Lung Squamous Cell Carcinom	3.395	KRAS	MYOCD low expression
TCGA-55-7911-01	TCGA	Primary Tumor	Lung	Lung Adenocarcinoma	3.374	KRAS	
TCGA-NJ-A4YI-01	TCGA	Primary Tumor	Lung	Lung Adenocarcinoma	3.33	KRAS	
TCGA-05-4395-01	TCGA	Primary Tumor	Lung	Lung Adenocarcinoma	3.314	KRAS	
TCGA-95-7567-01	TCGA	Primary Tumor	Lung	Lung Adenocarcinoma	3.251	KRAS	
TCGA-55-A490-01	TCGA	Primary Tumor	Lung	Lung Adenocarcinoma	3.209	KRAS	
TCGA-52-7812-01	TCGA	Primary Tumor	Lung	Lung Squamous Cell Carcinom	3.183	KRAS	
TCGA-05-4390-01	TCGA	Primary Tumor	Lung	Lung Adenocarcinoma	3.114	KRAS	
TCGA-95-7562-01	TCGA	Primary Tumor	Lung	Lung Adenocarcinoma	3.081	KRAS	
TCGA-MP-A4TE-01	TCGA	Primary Tumor	Lung	Lung Adenocarcinoma	2.941	KRAS	
TCGA-86-8054-01	TCGA	Primary Tumor	Lung	Lung Adenocarcinoma	2.934	KRAS	
TCGA-4B-A93V-01	TCGA	Primary Tumor	Lung	Lung Adenocarcinoma	2.82	KRAS	
TCGA-64-5774-01	TCGA	Primary Tumor	Lung	Lung Adenocarcinoma	2.791	KRAS	
TCGA-55-7815-01	TCGA	Primary Tumor	Lung	Lung Adenocarcinoma	2.718	KRAS	
TCGA-55-8615-01	TCGA	Primary Tumor	Lung	Lung Adenocarcinoma	2.683	KRAS	
TCGA-53-7813-01	TCGA	Primary Tumor	Lung	Lung Adenocarcinoma	2.448	KRAS	
TCGA-64-5778-01	TCGA	Primary Tumor	Lung	Lung Adenocarcinoma	2.374	KRAS	
TCGA-NJ-A4YP-01	TCGA	Primary Tumor	Lung	Lung Adenocarcinoma	2.337	KRAS	
TCGA-43-2581-01	TCGA	Primary Tumor	Lung	Lung Squamous Cell Carcinom	2.274	KRAS	
TCGA-55-A494-01	TCGA	Primary Tumor	Lung	Lung Adenocarcinoma	1.982	KRAS	

TCGA-O2-A52Q-01	TCGA	Primary Tumor	Lung	Lung Squamous Cell Carcinom	1.781	KRAS
TCGA-91-6836-01	TCGA	Primary Tumor	Lung	Lung Adenocarcinoma	1.721	KRAS
TCGA-55-8094-01	TCGA	Primary Tumor	Lung	Lung Adenocarcinoma	1.592	KRAS
TCGA-49-AARQ-01	TCGA	Primary Tumor	Lung	Lung Adenocarcinoma	0.9279	KRAS
TCGA-49-4506-01	TCGA	Primary Tumor	Lung	Lung Adenocarcinoma	0.7484	KRAS
TCGA-MF-A522-01	TCGA	Primary Tumor	Lung	Lung Squamous Cell Carcinom	0	KRAS

The clinical database is origin from UCSC Xena (http://xena.ucsc.edu/compare-tissue/).
MYOCD expression was analyzed, the median value was chosen as a cutoff.
Then analysis the percentage patients with low expression of MYOCD in patients with KRAS mutations.
